# E3 ubiquitin ligase NEURL3 promotes innate antiviral response through catalyzing K63‐linked ubiquitination of IRF7

**DOI:** 10.1096/fj.202200316R

**Published:** 2022-07-06

**Authors:** Fang Qi, Xin Zhang, Likun Wang, Caixia Ren, Xuyang Zhao, Jianyuan Luo, Dan Lu

**Affiliations:** ^1^ Institute of Systems Biomedicine School of Basic Medical Sciences Beijing Key Laboratory of Tumor Systems Biology Peking University Health Science Center Beijing P.R. China; ^2^ Department of Human Anatomy, Histology and Embryology Peking University Health Science Center Beijing P.R. China; ^3^ Department of Medical Genetics Peking University Health Science Center Beijing P.R. China

**Keywords:** antiviral immune response, E3 ubiquitin ligase, IRF7, NEURL3, viral infection

## Abstract

Interferon regulatory factor 7 (IRF7), as the interferon‐stimulated gene, maximally drives type I interferon (IFN) production. However, the mechanisms by which the biological function of IRF7 is regulated remain elusive. In this study, we found that IRF7 selectively interacted with the neuralized E3 ubiquitin‐protein ligase 3 (NEURL3). In concomitant with IRF7 induction, NEURL3 is upregulated by NF‐κB signaling in the late phase of viral infection. Moreover, NEURL3 augmented the host antiviral immune response through ubiquitinating IRF7. A mechanistic study revealed that NEURL3 triggered K63‐linked poly‐ubiquitination on IRF7 lysine 375, which in turn epigenetically enhanced the transcription of interferon‐stimulated genes (ISGs) through disruption of the association of IRF7 with Histone Deacetylase 1 (HDAC1), consequently augmenting host antiviral immune response. Accordingly, *Neurl3* ^*−/−*^ mice produced less type I IFNs and exhibited increased susceptibility to viral infection. Taken together, our findings identify NEURL3 as an E3 ubiquitin ligase of IRF7 and shed new light on the positive regulation of IRF7 in host antiviral immune signaling.

**Abbreviations**
HDAC1: histone deacetylase 1
IFN: interferon
IRF7: interferon regulatory factor 7
ISGs: interferon‐stimulated genes
LINCR: lung‐inducible neuralized‐related C3HC4 ring domain protein
MAVS: mitochondrial antiviral signaling
NEURL3: neuralized E3 ubiquitin‐protein ligase 3
PAMPs: pathogen‐associated molecular patterns
RLRs: RIG‐I‐like receptors
TLRs: toll‐like receptors

## 1 INTRODUCTION

Recognition of pathogen‐associated molecular patterns (PAMPs) by pattern recognition receptors (PRRs) represents the first step to trigger host innate immune signaling that leads to the production of the type I interferon (IFN) and other inflammatory cytokines.^1^ As the canonical PAMPs, foreign nucleic acids including RNAs and DNAs can be detected by the Toll‐like receptors (TLRs), RIG‐I‐like receptors (RLRs), and cytosolic DNA sensors.^2^, ^3^ Upon engagement with PAMPs, these PRRs recruit and oligomerize adaptor proteins such as TLR adaptor molecule 1 (TICAM1, also called TRIF), MyD88, mitochondrial antiviral signaling (MAVS), and Stimulator‐of‐interferon genes (STING), eventually resulting in the activation of TANK‐binding kinase 1 (TBK1) and IKKε.^4^, ^5^ These kinases subsequently phosphorylate a series of transcription factors, such as IFN regulator factor 3 (IRF3), IRF7 and nuclear factor kappa‐B (NF‐κB), to trigger nuclear translocation and initiate the transcriptional program of host innate immunity.^6^

In contrast to the ubiquitous expression of IRF3 and NF‐κB, the basal level of IRF7 is very low in resting mammalian cells that can be subsequently induced by type I IFN stimulation.^7^ In light of the substantial role of IRF7 in type I IFN production,^8^ it is conceivable that IRF7 plays a predominant role in the late stage of antiviral response. Among IRF family members, IRF3 and IRF7 share with greatest structural homology,^9^ suggesting their function can be regulated in a similar manner. For instance, the E3 ubiquitin ligase RAUL triggers the K48‐linked ubiquitination of both IRF3 and IRF7 and ultimately blocks IRF7‐mediated immune signaling by inducing degradation of IRF7.^10^ Similar to IRF3, IRF7 is activated by TRAF6 through induction of K63‐linked ubiquitination of IRF7, which is required prior to TBK1 or IKKε‐mediated phosphorylation.^11^ However, the regulatory mechanisms that specifically target IRF7 are largely unknown.

NEURL3 is also known as LINCR (Lung‐inducible Neuralized‐related C3HC4 RING domain protein), originally identified in mice to be induced in the alveolar epithelial cells upon exposure to lipopolysaccharide (LPS), which can be attenuated in the treatment with glucocorticoid.^12^, ^13^ In addition to inflammation, other studies also reveal that NEURL3 is implicated in lung development and spermatogenesis.^14^, ^15^ Moreover, loss of NEURL3 elicits stimulatory effects on hepatitis C virus (HCV) infection through targeting HCV glycoprotein E1.^16^ However, the biological function of NEURL3 in vivo remains elusive due to the lack of *Neurl3* knockout mouse model.

In this study, we reported that NEURL3 acted as the E3 ubiquitin ligase of IRF7. Through ubiquitination of the K63‐linked poly‐ubiquitin chain on IRF7 lysine 375, NEURL3 enhanced IRF7‐mediated transcription of ISGs by increasing the H3K27ac signal on the IRF7‐binding regions, thereby augmenting innate immune response. Accordingly, upon exposure to the virus, *Neurl3* ^*−/−*^ mice produced less type I IFNs and exhibited severe inflammatory damage. Our data thus identify the positive role of NEURL3 in host antiviral immune response and provide a new insight into the regulation of IRF7 biological function.

## 2 MATERIALS AND METHODS

### 2.1 Mice

To generate *Neurl3* ^*−/−*^ mice, E 0.5‐day mouse zygote injected with the mixture of 2.5 ng/μl guide RNA(CAGAGGGCTTCGTTCGTGCG) designed to insert a T in exon2 of *Neurl3* and 5 ng/μl spCas9 protein was cultured in KSOM medium overnight and transplanted into 0.5‐day pseudopregnant C57BL/6N female mice the next day. This results in the insertion of exon2 and loss of function of the *Neurl3* gene by generating a frameshift in all downstream exons. Chimeras were used for the generation of homozygous mice. *Neurl3* ^*+/−*^ mice were backcrossed with C57BL/6N WT mice for five generations. Mice were genotyped by sequencing the PCR products of DNA isolated from tails with the following primers: forward primer 5′‐AAGCCGGTGGGTTAGAATGG‐3′ and reverse primer 5′‐CTTTCGTGGTCCCGTACACA‐3′ and *Neurl3* ^*−/−*^ mice were produced by *Neurl3* ^*+/−*^ heterozygous mice after the backcross. All the mice were maintained under pathogen‐free conditions with the approval of the Ethics Committee of Peking University Health Science Center.

### 2.2 Cell lines

HEK‐293 T, HeLa, H1299, A549, SW480, Vero, NIH3T3, and MEF cells were obtained from American Type Culture Collection (ATCC). HEK‐293 T, Hela, Vero, NIH3T3, and MEF cells were cultured at 37°C under 5% CO_2_ in DMEM supplemented with 10% FBS and 1% penicillin/streptomycin. A549 cell line was cultured at 37°C under 5% CO_2_ in F12‐K supplemented with 10% FBS and 1% penicillin/streptomycin. H1299 and SW480 cell lines were cultured at 37°C under 5% CO_2_ in RPMI 1640 supplemented with 10% FBS and 1% penicillin plus streptomycin. H1299‐NEURL3 cells were generated by a lentivirus expression system in which lentiviral vector pcDH‐NEURL3‐FLAG‐IRES‐Puro or pcDH‐FLAG‐IRES‐Puro (empty vector) were co‐transfected with the packaging vector psPAX2 and envelope vector pMD2.G in HEK293T. Viral supernatants were collected to infect H1299 cells and then 2 μg/ml puromycin (ACROS) was added for 1 week until the H1299 cells without transfection were dead. Collected and expanded the living cells for identification and other assays.

### 2.3 Antibodies

The following commercial antibodies were used in this study: anti‐FLAG antibody (M2, F3165, Sigma–Aldrich), anti‐HA antibody (HA‐7, H3663, Sigma–Aldrich), anti‐GAPDH antibody (MC4, RM2002, Beijing Ray Antibody Biotech), anti‐β‐actin antibody (PM053, MBL), anti‐Pan Acetylation Monoclonal antibody (66 289, Proteintech), anti‐ubiquitin antibody (10201‐2‐AP, Proteintech), anti‐β‐actin antibody (PM053, MBL), anti‐IRF7 antibody (D8V1J #72073S, Cell Signaling Technology), anti‐IRF3 antibody (SL‐12, sc‐33 641, Santa Cruz), anti‐histone H3 (acetyl K27) antibody (ab4729, Abcam), and anti‐α‐tubulin antibody (MG17, RM2007, Beijing Ray Antibody Biotech).

To prepare the NEURL3 antibody, DNA corresponding to full length 1–262 amino acids of NEURL3 of human origin was subcloned into the expression vector pTriEx with a His‐tag and a sequence of MBP that helps to dissolve. The resultant protein was purified by histidine affinity chromatography in *E.coli* for antibody production in mouse. The specificity of the polyclonal antibody against NEURL3 was assessed by immunoblot.

### 2.4 Virus propagation and titration

Sendai virus (SeV) was propagated and amplified by inoculating a 10‐day‐old specific‐pathogen‐free chick embryo allantoic cavity. Vesicular stomatitis virus (VSV) and VSV‐GFP were propagated and amplified by infection of Vero cells. VSV/VSV‐GFP and SeV were titrated to determine the TCID50 in L929 cells and A549 cells, respectively.

### 2.5 Virus infection in vitro and in vivo

For in vitro virus infection studies, a series of cell lines including H1299, MEFs, NIH3T3, HEK293T, A549, or SW480 were plated 24 h before infection. Cells were infected with VSV (0.1 MOI), VSV‐GFP (0.1 MOI) or SeV (1 MOI) for indicated time. For in vivo virus infection studies, age‐ and sex‐matched (6–8 weeks old) WT and *Neurl3* ^*−/−*^ littermate mice were intraperitoneally infected with SeV (5 × 10^5^ PFU/mouse) and intravenously infected with VSV (8 × 10^7^ PFU/mouse). For the survival experiments, mice were monitored for survival after VSV infection. VSV genomic DNA copy number and cytokine production in liver and lung were determined by real‐time PCR at 3 days post‐infection. Livers from VSV‐infected mice were dissected. A part of them was fixed in 10% phosphate‐buffered formalin, embedded into paraffin, sectioned, stained with hematoxylin–eosin solution, and examined by light microscopy for histological changes. The other part of them were lysed in RIPA lysis buffer and completed for quantitative proteomics by mass spectrometry after the determination of protein concentration.

### 2.6 Immunofluorescence staining and microscopy

HeLa cells were grown on coverslips and transfected with the indicated plasmids. At 24 h after transfection, cells were washed once with PBS and then were fixed for 15 min with 4% PFA and permeabilized for 30 min with 0.5% (v/v) Triton X‐100/PBS. After incubation for blockade of 1% (w/v) BSA dissolved in PBS, primary antibodies were incubated for 1 h at room temperature, followed by staining with fluorophore‐conjugated secondary antibody (555– or 488– conjugated) for 40 min at room temperature and DAPI (BioDee Biotechnology) was used to indicate nucleus. Coverslips were mounted and assessed with fluorescence microscopy. Images were acquired on a Nikon TCS A1 microscope and NIS Elements Viewer 4.20 software was used to analyze the confocal assay.

### 2.7 Flow cytometry

H1299 cells were washed with PBS and re‐suspended in PBS with 1% fetal bovine serum (FBS) after trypsinization. Single‐cell suspensions were processed for flow cytometry using the flow cytometry analyzer (BD Biosciences) with FACSuite Software Bundle v1.0 (BD Biosciences). The FACS data were analyzed with FlowJo v7.6.1 software.

### 2.8 Quantitative real‐time PCR (qRT‐PCR)

Total RNA from tissues or cells were extracted with TRIzol reagent (Invitrogen) according to the instruction and reverse transcribed into cDNA with the GoScript Reverse Transcription System (Promega). QRT‐PCR was carried out using TransStart Top Green qPCRSuperMix (TransGen Biotech). Reverse‐transcription products of samples were amplified by Applied Biosystems 7500 Fast & 7500 Real‐Time PCR System and analyzed by 7500 Software v2.3 (Applied Biosystems) using TransStart Top Green qPCR SuperMix (TransGen Biotech) according to the manufacturer's instructions. Primers used for qRT‐PCR assays were listed in Table S1.

### 2.9 Pull‐down assay and mass spectrometry

HEK293T cells transfected with FLAG‐tagged‐IRF7 were lysed in NP‐40 lysis buffer (150 mM NaCl, 1 mM EDTA, 20 mM Tris–HCl, pH 8.0, 10% (v/v) glycerol, 0.5% (v/v) NP40) with protein inhibitor cocktail (Roche) and PMSF. The whole‐cell extracts were incubated with anti‐FLAG M2 Affinity Gel (Sigma Aldrich) for 4 h at 4°C and precipitants were washed three times using PBS with 0.1% (v/v) NP40. The pull‐down products were eluted by 3 × FLAG‐peptide (Sigma–Aldrich) and subjected to SDS‐PAGE. The whole page was stained by Pierce Silver Stain for Mass Spectrometry Kit (Thermo Scientific) and evaluated with mass spectrometry assay.

### 2.10 Immunoblot and coimmunoprecipitation assay

For immunoblot analysis, cells or tissues were lysed with NP‐40 lysis buffer supplemented with a protease inhibitor cocktail. Protein concentrations in the extracts were measured with a BCA Protein Assay Kit (Thermo Scientific) and were made equal with the extraction reagent. For co‐immunoprecipitation, whole‐cell extracts were collected 24 h after transfection and were lysed in NP‐40 lysis buffer supplemented with a protease inhibitor cocktail. Lysates were centrifuged and incubated with Protein A/G beads (EMD Millipore) at 4°C together with 1 μg corresponding antibodies. After 4 h incubation, beads were washed three times using PBS with 0.1% (v/v) NP40. Immunoprecipitants were eluted by boiling with 1% (w/v) SDS sample buffer for immunoblot analysis.

### 2.11 Native PAGE

The IRF3 dimerization assay was performed as described.^17^ HEK293T cells were transfected with indicated plasmids and harvested with 100 μl ice‐cold lysis buffer (50 mM Tris–HCl, pH 7.5; 150 mM NaCl and 0.5% NP‐40 containing protease inhibitor cocktail). Supernatant protein was quantified and diluted with 2 × native PAGE sample buffer (125 mM Tris–HCl, pH 6.8; 30% glycerol and 0.1% bromophenol blue), followed by 8% native polyacrylamide gel for separation.

### 2.12 Luciferase reporter assay

HEK293T cells were transfected with pGL3‐*IFNβ* or pGL3‐*ISRE* luciferase reporter plasmid and a control pRL‐TK renilla luciferase reporter, together with vector encoding various proteins. The details of the transfection system were performed as previously described.^18^ For analysis of the effect of p65 on NEURL3 promoter activity, HEK293T cells were transfected with the pGL3‐NEURL3 luciferase reporter plasmid and a control pRL‐TK renilla luciferase reporter together with vector encoding p65 at various concentrations. Cells were processed using Dual‐Luciferase Reporter Assay System (Promega) as per the manufacturer's instruction. Firefly luciferase expression was normalized to *Renilla* luciferase expression.

### 2.13 Ubiquitination assay

For analysis of the ubiquitination of IRF7, HEK293T cells were transfected with FLAG‐IRF7 WT (or mutants), HA‐ubiquitin WT (or mutants), and S‐tag‐HA‐NEURL3 WT (or mutants), then whole‐cell extracts were immunoprecipitated with anti‐FLAG antibody and analyzed by immunoblot with anti‐HA antibody. The transfection system of S‐tag‐HA‐IRF7, HA‐ubiquitin, and FLAG‐NEURL3 was also used to analyze the ubiquitination of IRF7 in HEK293T, and then whole‐cell extracts were immunoprecipitated with S‐tag agarose and analyzed by immunoblot with anti‐HA and anti‐FLAG antibodies.

### 2.14 Nuclear‐cytoplasmic fractionation

Immunoblot analysis of HA‐IRF7 in nuclear and cytoplasmic fractions in HEK293T cells transfected with HA‐IRF7‐WT/K375R, followed by VSV infection according to the manufacturer's instructions for Nuclear‐Cytosol Extraction Kit (APPLYGEN P1200).

### 2.15 Chromatin immunoprecipitation‐qPCR (ChIP‐qPCR)

Chromatin immunoprecipitation assay was performed as described previously.^18^ Briefly, 1 × 10^7^ HEK293T cells transfected with plasmids were crosslinked with 1% formaldehyde for 10 min at room temperature and quenched by 125 mM Glycine. Cells were washed three times with PBS and resuspended in 0.3 ml of ChIP lysis buffer (50 mM Tris–HCl (pH 8.0), 5 mM EDTA, 1% SDS, supplemented with cOmplete™ protease inhibitor cocktail (Roche), and 1 mM PMSF). Chromatin was sheared by sonication to 200–1000 bp fragments. Then, one‐third of chromatin was removed and saved as input. The residual chromatin was diluted with ninefold ChIP dilution buffer (20 mM Tris–HCl (pH 8.0), 150 mM NaCl, 2 mM EDTA, 1% Triton X‐100, supplemented with cOmplete™ protease inhibitor cocktail (Roche), and 1 mM PMSF). Half the dilution chromatin was added with 2 μg rabbit polyclonal anti‐histone H3 (acetyl K27) antibody (ab4729, Abcam), and a half was added with 2 μg control IgG (normal rabbit IgG, sc‐2027, Santa Cruz), followed by incubation at 4°C overnight. Next, 40 μl of Dynabeads Protein G (Invitrogen) was added and incubated at 4°C for 4 h. Beads bound with chromatin were washed sequentially with TSE‐I buffer (20 mM Tris–HCl (pH 8.0), 150 mM NaCl, 2 mM EDTA, 1% Triton X‐100, 0.1% SDS), TSE‐II buffer (20 mM Tris–HCl (pH 8.0), 500 mM NaCl, 2 mM EDTA, 1% Triton X‐100, 0.1% SDS), LiCl wash buffer III (10 mM Tris–HCl (pH 8.0), 1 mM EDTA, 1% NP40, 1% sodium deoxycholate, 0.25 M LiCl) and TE buffer (10 mM Tris–HCl (pH 8.0), 1 mM EDTA) for 10 min at 4°C. DNA was eluted from the beads with 100 μl elution buffer (1% SDS, 0.1 M NaHCO3) at room temperature for 10 min. Then, NaCl (final 200 mM) was added to the elution and input samples, followed by incubation at 65°C overnight to reverse the formaldehyde cross‐link. Subsequently, 50 μg/ml RNase A (TIANGEN Biotech) was added, followed by incubation at 37°C for 30 min. 50 μg/ml Proteinase K (Zoman Biotechnology), 10 mM EDTA, and 40 mM Tris–HCl (pH 8.0) were added, followed by incubation at 55°C for 1 h. Extracted DNA with extracted QIAquick PCR Purification Kit (Qiagen). Quantitative real‐time PCR was performed with specific primers (Table S2).

### 2.16 Bioinformatics

The expression profile of NEURL3 was interrogated from THE HUMAN PROTEIN ATLAS.^19^ For gene‐set enrichment analysis (GSEA) of liver's quantitative proteomics, differentially expressed proteins were analyzed using applications from Broad Institute 21 (http://www.broad.mit.edu/gsea/software/software_index.html) with default parameters.

### 2.17 Statistical analysis

GraphPad Prism 7.00 software was used for analysis and all data were presented as means ± SD. The statistical significance between the two groups was calculated with a two‐tailed unpaired Student's *t* test and log‐rank (Mantel‐Cox) test for mouse survival studies, with *p* < .05 considered statistically significant.

## 3 RESULTS

### 3.1 IRF7 physically interacts with NEURL3

**FIGURE 1:**
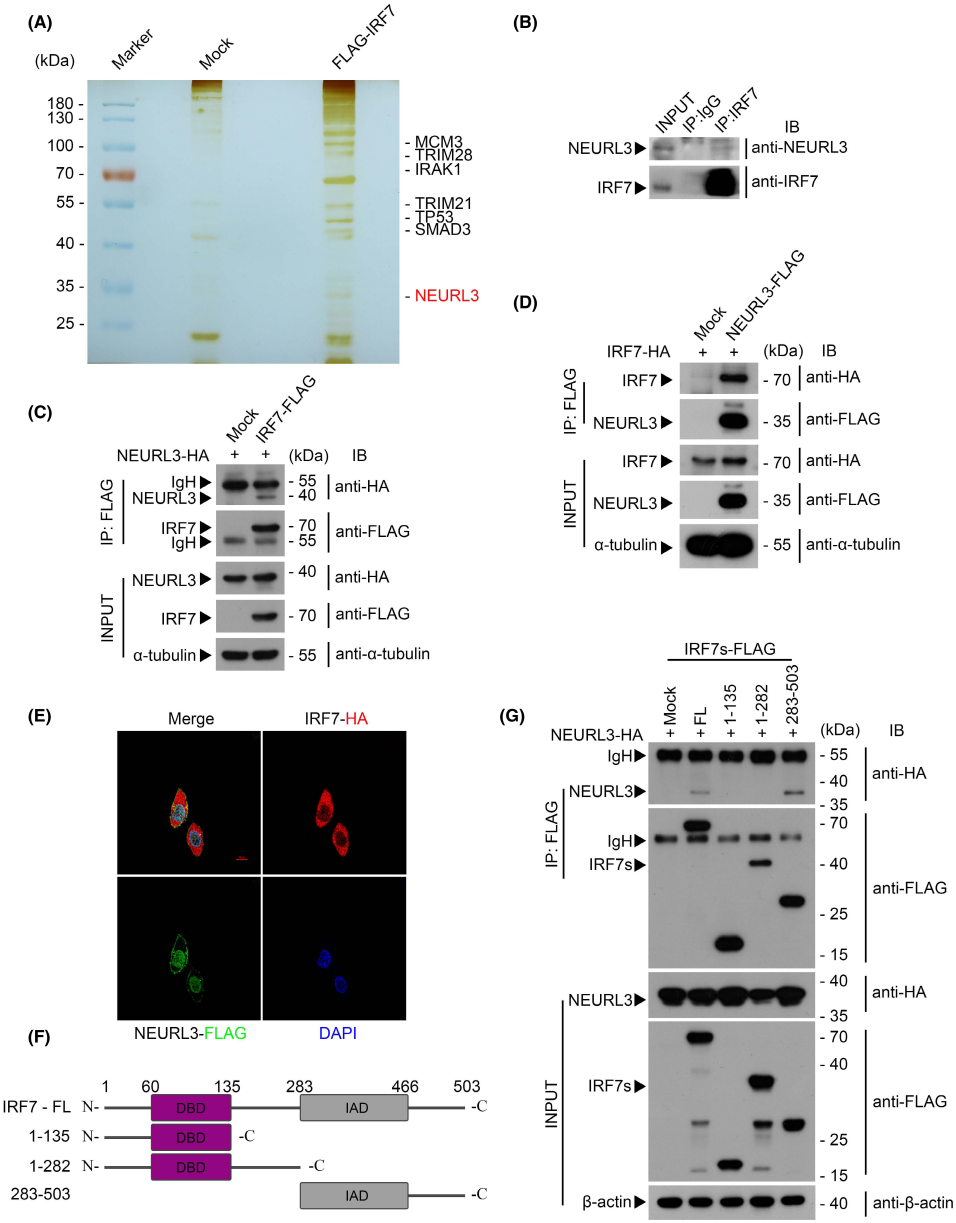
IRF7 interacts with the E3 ubiquitin ligase NEURL3. (A) Silver staining and mass spectrometry (MS) analysis of IRF7‐associated proteins after immunoprecipitation (with anti‐FLAG beads) from lysates of HEK293T cells transfected with FLAG‐IRF7 or empty vector and subsequently infected with VSV for 8 h. (B) Immunoprecipitation of endogenous IRF7 from lysates of virus‐infected murine liver tissue with an anti‐IRF7 monoclonal antibody or rabbit IgG and then subjected to western blotting with anti‐NEURL3 antibody and anti‐IRF7 antibody. (C) Coimmunoprecipitation analysis of HEK293T cells transfected with FLAG‐IRF7 or empty vector and HA‐NEURL3; lysates immunoprecipitated with anti‐FLAG antibody were analyzed by immunoblot with anti‐HA antibody. α‐tubulin was used as a loading control. IP, immunoprecipitation. (D) Coimmunoprecipitation analysis of HEK293T cells transfected with FLAG‐NEURL3 or empty vector and HA‐IRF7; lysates immunoprecipitated with anti‐FLAG antibody were analyzed by immunoblot with anti‐HA antibody. α‐tubulin was used as a loading control. IP, immunoprecipitation. (E) Anti‐FLAG and anti‐HA specific immunostaining of Hela cells transfected with HA‐IRF7 and FLAG‐NEURL3. The interaction was shown by confocal fluorescence microscopy. DAPI was used to indicate the nucleus. Scale bars, 10 μm. (F) Schematic representation of full‐length IRF7 and IRF7 truncation mutants. IRF7‐1‐135 (from N terminal to DBD domain), IRF7‐1‐282 (IAD domain and C terminal linker deleted), IRF7‐283‐503 (from IAD domain to C terminal). (G) Coimmunoprecipitation analysis of HEK293T cells transfected with FLAG‐IRF7 truncation mutants or empty vector and HA‐NEURL3; lysates immunoprecipitated with anti‐FLAG antibody were analyzed by immunoblot with anti‐HA antibody. β‐actin was used as a loading control. Mock, empty vector; FL, full‐length IRF7; 1–135, IRF7 of 1–135 amino acid; 1–282, IRF7 of 1–282 amino acid; 283–503, IRF7 of 283–503 amino acid.

IRF7 is critical for the host innate immune response against viral infection.^8^ To identify potential regulatory mechanisms of IRF7 activation, we transfected the plasmids encoding IRF7 or Mock into HEK293T cells, which were subsequently infected with VSV for 8 h. Following the application of affinity purification with anti‐FLAG beads, the interactome of IRF7 was analyzed by mass spectrometry assay. As shown in Figure 1A, the E3 ubiquitin ligase NEURL3 was identified as a strong IRF7 binding partner during viral infection. Ensued co‐immunoprecipitation assay and Con‐focal assay were performed to further confirm the relationship between IRF7 and NEURL3 (Figure 1B–E). To determine whether other members of the IRF family interacted with NEURL3, we transfected vector encoding NEURL3 into HEK293T cells together with vector encoding IRF family members (IRF1‐9). Co‐immunoprecipitation assays showed that with the exception of IRF3 and IRF7, all other IRFs hardly interacted with NEURL3 (Figure S1A–C). We next co‐expressed NEURL3‐HA with a series of truncated forms of IRF7 to map the NEURL3‐binding region of IRF7 and our results revealed that the C‐terminal domain of IRF7 was essential for its interaction with NEURL3 (Figure 1F,G). In addition, we found that the N‐terminal of NEURL3 was required for its physical association with IRF7, as shown by reciprocal precipitation experiments between IRF7 and a series of truncations of NEURL3 (Figure S1D,E). Our results thus demonstrate that IRF7 selectively interacts with NEURL3 during viral infection.

### 3.2 NEURL3 is upregulated in the late phase of viral infection

**FIGURE 2:**
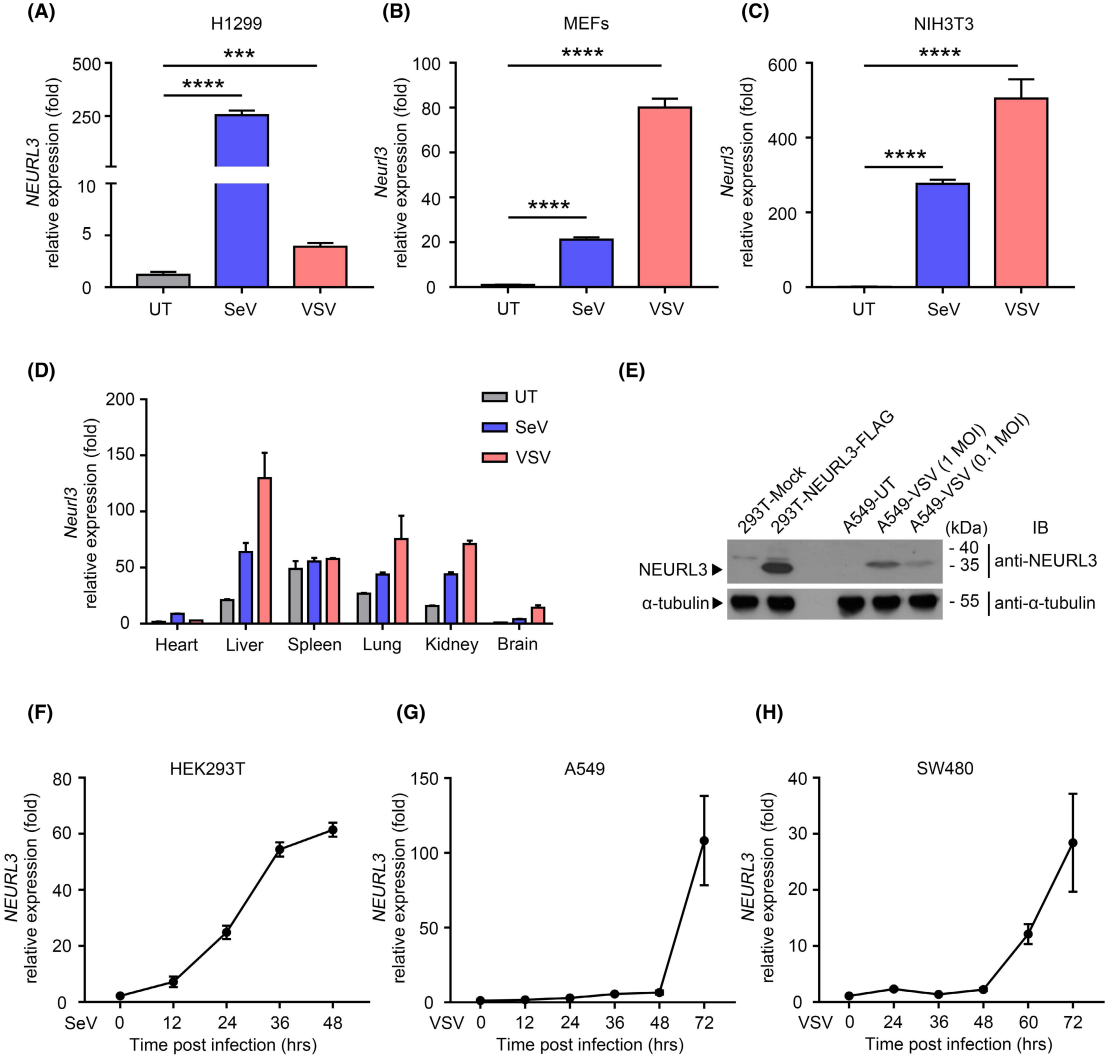
Viral infection stimulates NEURL3 expression. (A–C) Expression of *NEURL3/Neurl3* mRNA in H1299, MEFs, and NIH3T3 cell lines infected with SeV (1 MOI) or VSV (0.1 MOI) for 24 h by qRT‐PCR analysis. UT, untreatment. (D) Expression of *Neurl3* mRNA in heart, liver, spleen, lung, kidney, and brain from 8‐week‐old C57BL/6 mice infected with SeV (5 × 10^5^ PFU/mouse) or VSV (8 × 10^7^ PFU/mouse) for 24 h. UT, untreatment. (E) Immunoblot analysis of NEURL3 protein expression in A549 cells upon infection with VSV (1 MOI or 0.1 MOI) for 24 h. The left two lines were used as the positive control transfected with FLAG‐NEURL3 or empty vector (Mock) in HEK293T cells. α‐tubulin was used as the loading control. A549‐UT, untreatment. (F–H) Expression of *NEURL3* mRNA in HEK293T, A549, and SW480 cell lines infected with SeV (1 MOI) or VSV (0.1 MOI) for gradient times post‐infection by qRT‐PCR analysis. Data shown in (A–C) are mean ± SD, ***p < .001, ****p < .0001, and statistical significance was assessed by a two‐tailed unpaired Student's *t* test.

Through interrogation of the expression profile of NEURL3 from the ProteinAtlas database (Human Protein Atlas proteinatlas.org),^19^ we found that NEURL3 is maintained lower level under a physiological condition in multiple tissues with exception of the pancreas and salivary gland (Figure S2). To assess the status of NEURL3 during viral infection, we used a qRT‐PCR assay and found that NEURL3 was induced in H1299 (Human lung cancer cell line), NIH3T3 (murine fibroblast cell line), and MEFs (murine embryonic fibroblast cell line) during Vesicular stomatitis virus (VSV), or Sendai virus (SeV) infection (Figure 2A–C). Interestingly, in contrast to the preferential sensitivity of NEURL3 induction in H1299 cell upon exposure to SeV, VSV infection triggered a higher level of NEURL3 in NIH3T3 and MEFs than SeV infection did (Figure 2A–C). Moreover, we performed qRT‐PCR experiments to assess the expression of Neurl3 in the tissues of C57BL/6 mice with or without viral infection. As shown in Figure 2D, inducible NEURL3 was detected in the liver, lung, brain, and kidney during viral infection. In addition to mRNA level, the increased protein level of NEURL3 was detected in the A549 lung cancer cell line upon VSV infection (Figure 2E). Notably, the remarkable induction of NEURL3 mainly occurred at 12, 24, and even 48 h post‐viral infection, which was considered the late phase of viral infection (Figure 2F–H). Given that IRF7 plays a predominant role in the late phase of viral infection, it is conceivable that NEURL3 preferentially regulates the activity of IRF7 rather than IRF3.

### 3.3 NEURL3 enhances innate immune response and limits viral replication

**FIGURE 3:**
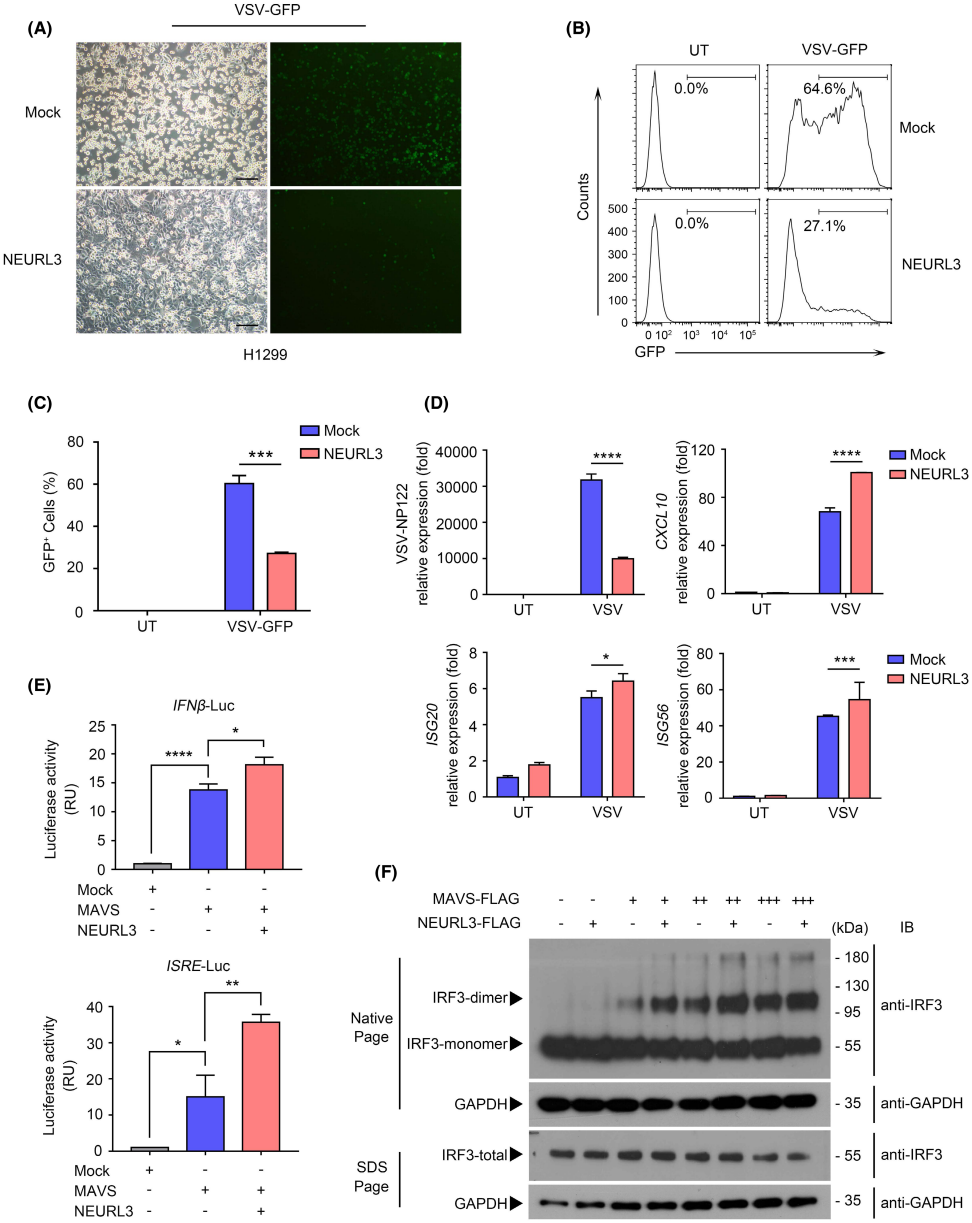
NEURL3 positively regulates host antiviral response. (A–D) H1299 cell lines stably expressing NEURL3 or empty vector were infected with VSV‐GFP at 0.1 MOI for 24 h. VSV‐encoded GFP expression was examined by imaging (A), flow cytometry (B), statistical analysis (C), and expression of VSV‐NP122 and ISGs mRNA. Data shown in (A–D) are all representative of three independent experiments. UT, untreatment. (A) The VSV‐GFP^+^ cells were observed through fluorescence microscopy and images were shown. Scale bars, 50 μm. (B and C) The percentage of virus‐infected cells (GFP^+^) detected by flow cytometry as shown in (B). Mean ± SD was plotted on the graph (C). (D) The mRNA levels of VSV‐NP122, *CXCL10*, *ISG20*, and *ISG56* were assessed by qRT‐PCR analysis. (E) Luciferase (Luc) activity in HEK293T cells transfected with *IFN‐β/ISRE* luciferase reporter together with MAVS or MAVS plus NEURL3 for 24 h. Mock, empty vector. (F) Native PAGE immunoblot analysis of IRF3 dimerization in HEK293T transfected with different amounts of FLAG‐MAVS in the presence or absence of FLAG‐NEURL3. GAPDH was used as a loading control. Data shown in (C–E) are mean ± SD, *p < .05, **p < .01, ***p < .001, ****p < .0001 and statistical significance was assessed by a two‐tailed unpaired Student's *t* test.

To investigate the role of NEURL3 in the host antiviral immune response, we overexpressed NEURL3 in H1299 cells that express extremely low levels of endogenous NEURL3 under quiescent conditions (Figure S3). Upon exposure to VSV‐GFP, less amount of GFP^+^ cells was detected in presence of NEURL3 as compared with control cells (Figure 3A–C). Consistent with these findings, the viral titration was also reduced in NEURL3‐expressing cells as detected by the qRT‐PCR assay (Figure 3D). Along with the reduction of viral titration, the expression of interferon‐stimulated genes such as *ISG20*, *ISG56*, and *CXCL10* was upregulated in NEURL3‐expressing cells (Figure 3D). To further determine that NEURL3 can enhance host innate immune response, we co‐transfected MAVS with NEURL3 into HEK293T cells. As shown in Figure 3E, both the interferon (IFN)‐luciferase reporter and IFN‐stimulated response element (ISRE)‐luciferase reporter assays showed that NEURL3 augmented MAVS‐mediated innate immune response in a dose‐dependent manner. In accordance with the luciferase assay, we also found that overexpression of NEURL3 enhanced MAVS function and promoted IRF3 dimerization assessed by the Native PAGE assay (Figure 3F). Our data thus indicate that NEURL3 can facilitate host immune response against viral infection.

### 3.4 NEURL3 acts as an E3 ubiquitin ligase of IRF7

**FIGURE 4:**
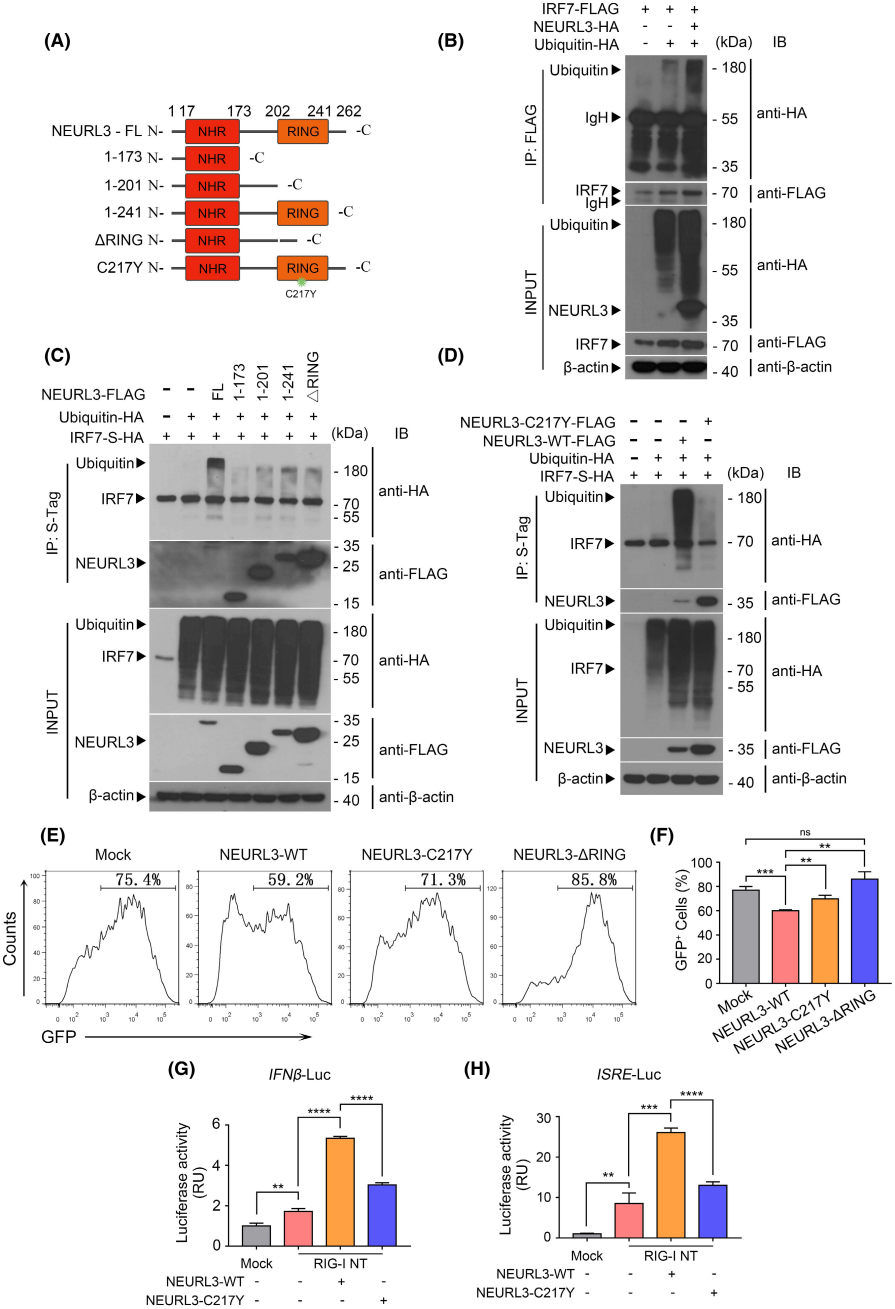
NEURL3 modulates IRF7 activity in an E3 ligase‐dependent manner. (A) Schematic representation of full‐length NEURL3 and a series of NEURL3 truncations. NEURL3‐1‐173 (from N terminal to NHR domain), NEURL3‐1‐241 (C terminal linker deleted), NEURL3‐174‐262 (N terminal linker and NHR domain deleted), NEURL3‐ΔRING (RING domain deleted), NEURL3‐C217Y (replacement of the cysteine residue with tyrosine). (B) Coimmunoprecipitation analysis of IRF7 ubiquitination in HEK293T cells transfected with FLAG‐IRF7 and HA‐ubiquitin in the presence of empty vector or HA‐NEURL3. Lysates immunoprecipitated with anti‐FLAG antibody were analyzed by immunoblot with anti‐HA antibody. β‐actin was used as a loading control. IP, immunoprecipitation. (C) Coimmunoprecipitation analysis of IRF7 ubiquitination in HEK293T cells transfected with S‐Tag‐HA‐IRF7 and HA‐ubiquitin in the presence of empty vector or FLAG‐NEURL3 full length or different FLAG‐NEURL3 truncation. Lysates immunoprecipitated with S‐Tag were analyzed by immunoblot with anti‐FLAG and anti‐HA antibodies, respectively. β‐actin was used as a loading control. IP, immunoprecipitation. (D) Coimmunoprecipitation analysis of IRF7 ubiquitination in HEK293T cells transfected with S‐Tag‐HA‐IRF7 and HA‐ubiquitin in the presence of empty vector or FLAG‐NEURL3‐WT or FLAG‐NEURL3‐C217Y mutation. Lysates immunoprecipitated with S‐Tag were analyzed by immunoblot with anti‐FLAG and anti‐HA antibodies, respectively. β‐actin was used as a loading control. IP, immunoprecipitation. (E and F) HEK293T transfected with empty vector (Mock), FLAG‐NEURL3‐WT, FLAG‐NEURL3‐C217Y, FLAG‐NEURL3‐ΔRING after infection with VSV‐GFP at 0.1 MOI for 24 h. VSV‐encoded GFP expression was examined by flow cytometry (E). Statistical analysis of the percentage of virus‐infected cells (GFP^+^) assessed by flow cytometry as shown in (F). Mean ± SD was plotted on the graph (F). (G and H) Luciferase (Luc) activity in HEK293T cells transfected with *IFN‐β/ISRE* luciferase reporter together with plasmids encoding RIG‐I NT or RIG‐I NT and NEURL3‐WT/NEURL3‐C217Y for 24 h. Mock, empty vector. NT, N terminal. Data shown in (F–H) are mean ± SD, ns *p* > .05, **p < .01, ***p < .001, ****p < .0001 and statistical significance was assessed by a two‐tailed unpaired Student's *t* test.

Considering that NEURL3 is a putative E3 ubiquitin ligase,^12^ we, therefore, tested the possibility that NEURL3 modulates host innate immune response by influencing post‐translational modification of IRF7. We co‐transfected full‐length IRF7 with Ub‐HA into HEK293T cell in the presence or absence of NEURL3 (Figure 4A). As shown in Figure 4B, ectopic expression of NEURL3 induced IRF7 ubiquitination markedly. To determine whether the antiviral effects of NRUEL3 depend on its E3 ubiquitin ligase activity, we cloned vectors encoding NEURL3 with deletion of its RING domain or mutant NEURL3^C217Y^ (Figure 4A). In light of the essential role of the RING domain in ubiquitin ligase activity, it is conceivable that deletion or inactivation of the RING domain will impair the E3 ubiquitin ligase activity of NEURL3. We next employed a co‐immunoprecipitation assay to further confirm these results. Compared with the increased ubiquitination of IRF7 by full‐length NEURL3, both deletion of RING domain and C217Y mutation hardly triggered IRF7 ubiquitination (Figure 4C,D). We next transfected these vectors into HEK293T cells respectively and followed infection with VSV‐GFP. As shown in Figure 4E,F, compared with the remarkable antiviral effects of full‐length NEURL3, the percentage of virus‐infected (GFP^+^) cells was increased in cells transfected with the vectors encoding NEURL3 with deletion of its RING domain or mutant NEURL3^C217Y^. Similar results were also detected by luciferase reporter assay (Figure 4G,H), which further confirms that NEURL3 exerts stimulatory effects on host antiviral immune response through ubiquitinating IRF7.

### 3.5 NEURL3 ubiquitinates the K63‐linked poly‐ubiquitin chain on IRF7 lysine 375

**FIGURE 5:**
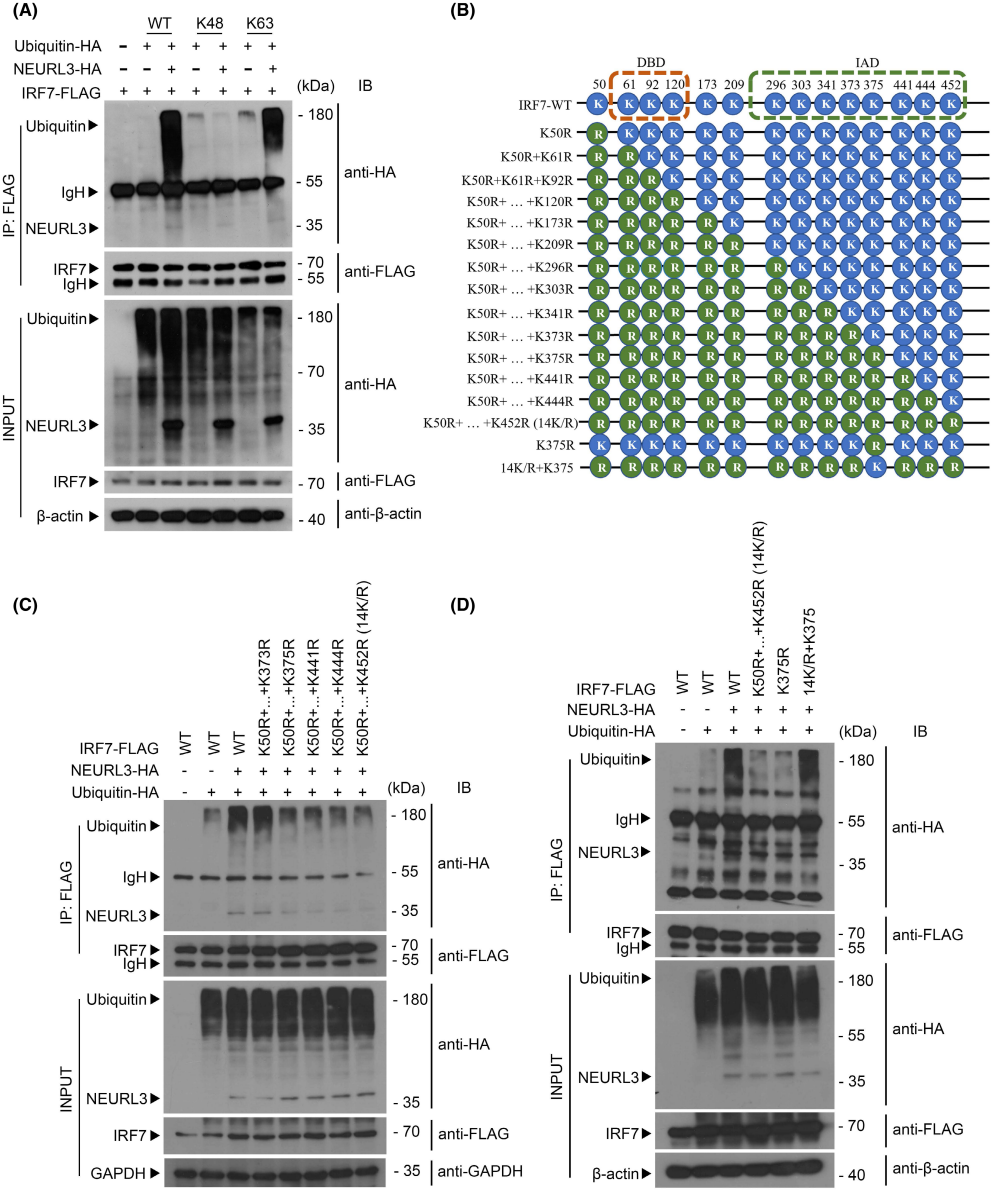
NEURL3 promotes K63‐linked ubiquitination of IRF7 at K375. (A) Coimmunoprecipitation analysis of IRF7 ubiquitination in HEK293T transfected with FLAG‐IRF7, HA‐NEURL3 or empty vector and HA‐ubiquitin‐WT, HA‐ubiquitin‐K48 or HA‐ubiqutin‐K63. Lysates immunoprecipitated with anti‐FLAG antibody were analyzed by immunoblot with anti‐HA antibody. β‐actin was used as a loading control. IP, immunoprecipitation. (B) Schematic representation of IRF7 WT and IRF7 mutations involved in Figures 5 and S4. (C) Coimmunoprecipitation analysis of IRF7 ubiquitination in HEK293T transfected with HA‐NEURL3 or empty vector, HA‐ubiquitin and FLAG‐IRF7‐WT, FLAG‐IRF7‐K50R + … + K373R, FLAG‐IRF7‐K50R + … + K375R, FLAG‐IRF7‐K50R + … + K441R, FLAG‐IRF7‐K50R + … + K444R, FLAG‐IRF7‐K50R + … + K452R. Lysates immunoprecipitated with anti‐FLAG antibody were analyzed by immunoblot with anti‐HA antibody. GAPDH was used as a loading control. IP, immunoprecipitation. (D) Coimmunoprecipitation analysis of IRF7 ubiquitination in HEK293T transfected with HA‐NEURL3 or empty vector, HA‐ubiquitin and FLAG‐IRF7‐WT, FLAG‐IRF7‐14KR, FLAG‐IRF7‐K375R, FLAG‐IRF7‐14KR + K375. Lysates immunoprecipitated with anti‐FLAG antibody were analyzed by immunoblot with anti‐HA antibody. β‐actin was used as a loading control. IP, immunoprecipitation.

To determine which kind of ubiquitin modification was triggered by NEURL3, we used a series of mutants of HA‐tagged ubiquitin in which remained only one of the seven lysine sites (K6, K11, K27, K29, K33, K48, and K63) or just the N‐terminal methionine (K0). Through the co‐immunoprecipitation assay, we found that NEURL3 predominantly induced the K63‐linked poly‐ubiquitination of IRF7 (Figures 5A and S4A,B). To identify the lysine residues on IRF7 that are specific ubiquitinated by NEURL3, we mutated the 14 lysine (K) in the IRF7 to arginine (R) one by one (Figure 5B). As shown in Figures 5C and S4C,D, the K375R mutation significantly impaired the stimulatory effects of NEURL3 on IRF7 ubiquitination. To determine whether K375 residue was required for NEURL3‐mediated ubiquitination of IRF7, we constructed a series of vectors encoding IRF7 with 14 K/R, IRF7 with only K375R and IRF7 with 13 K/R exception of K375, respectively (Figure 5B). As shown in Figure 5D, in contrast to the remarkable ubiquitination of wild‐type IRF7 by NEURL3, little modification was detected in IRF7 with 14 K/R. Similar to IRF7 with 14 K/R, NEURL3 exerted little effect on the ubiquitination of the single mutation (K375R) (Figure 5D). Reciprocally, NEURL3 triggered ubiquitination of IRF7 with 13 K/R exceptions of K375 (Figure 5D). Our data thus demonstrate that NEURL3 selectively ubiquitinates the K63‐linked poly‐ubiquitin chain on IRF7 lysine 375.

### 3.6 Ubiquitination on lysine 375 of IRF7 enhances its transcriptional activity

**FIGURE 6:**
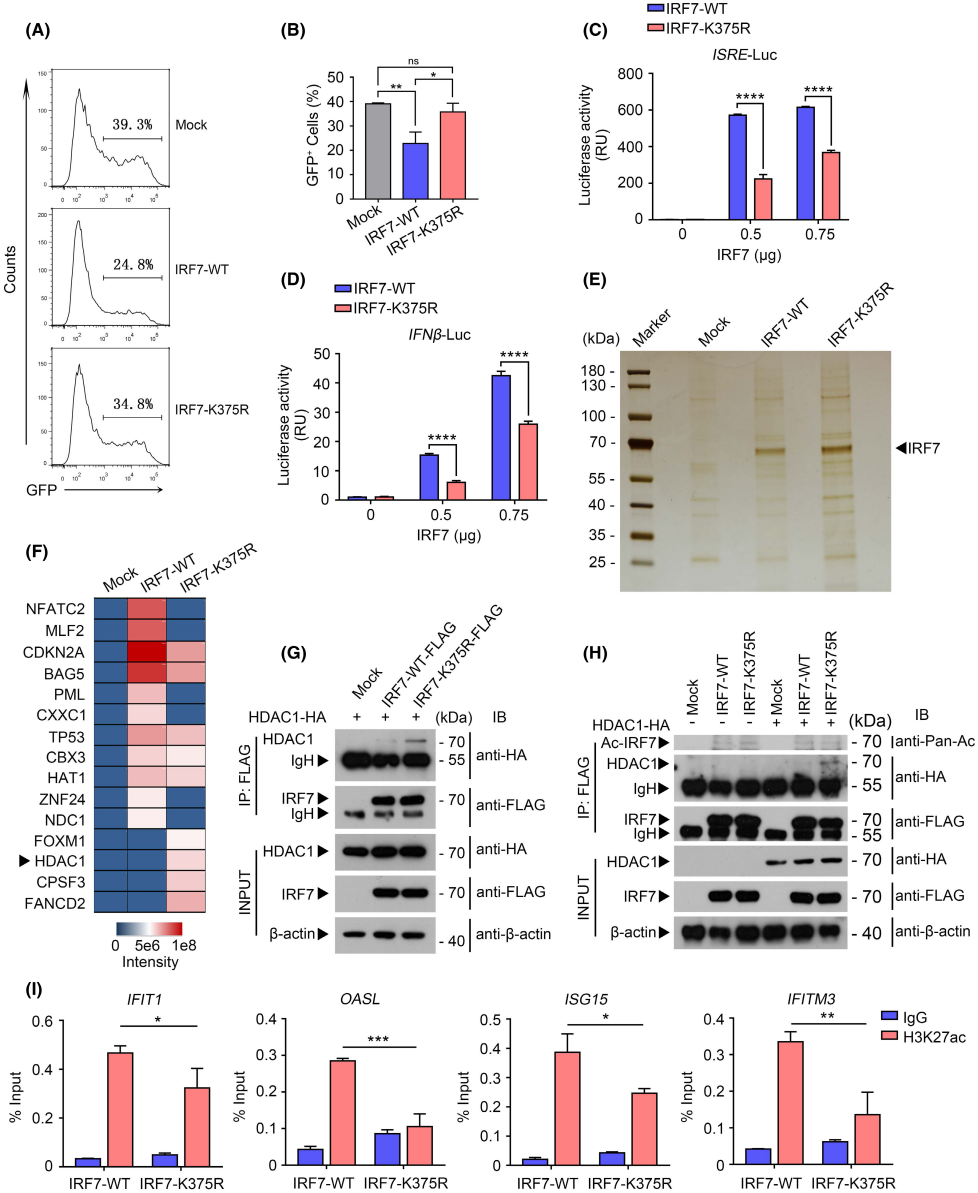
The ubiquitination of IRF7 at K375 facilitates its transcriptional activity. (A and B) HEK293T transfected with empty vector (Mock), IRF7‐WT, IRF7‐K375R after infection with VSV‐GFP at 0.1 MOI for 24 h. VSV‐encoded GFP expression was examined by flow cytometry (A). Statistical analysis of the percentage of virus‐infected cells (GFP^+^) by flow cytometry as shown in (B). Mean ± SD was plotted on the graph (B). (C) Luciferase (Luc) activity in HEK293T cells transfected with *ISRE* luciferase reporter together with plasmids encoding IRF7‐WT or IRF7‐K375R in different doses (0, 0.5, 0.75 μg) for 24 h. (D) Luciferase (Luc) activity in HEK293T cells transfected with *IFN‐β* luciferase reporter together with plasmids encoding IRF7‐WT or IRF7‐K375R in different doses (0, 0.5, 0.75 μg) for 24 h. (E) Silver staining and mass spectrometry (MS) analysis of IRF7‐WT or IRF7‐K375R associated proteins after immunoprecipitation (with anti‐FLAG beads) from lysates of HEK293T cells transfected with FLAG‐IRF7‐WT, FLAG‐IRF7‐K375R or empty vector and subsequently infected with VSV for 8 h. (F) Mass spectrometry (MS) analysis of IRF7‐WT or IRF7‐K375R associated proteins in HEK293T infected with VSV for 8 h. The relative expression of associated proteins was calculated by the change‐in‐threshold method. (G) Coimmunoprecipitation analysis of HEK293T cells transfected with FLAG‐IRF7‐WT, FLAG‐IRF7‐K375R or empty vector and HA‐HDAC1; lysates immunoprecipitated with anti‐FLAG antibody were analyzed by immunoblot with anti‐HA antibody. β‐actin was used as a loading control. IP, immunoprecipitation. (H) Coimmunoprecipitation analysis of IRF7 acetylation in HEK293T cells transfected with FLAG‐IRF7‐WT, FLAG‐IRF7‐K375R or empty vector with or without HA‐HDAC1. Lysates immunoprecipitated with anti‐FLAG antibody were analyzed by immunoblot with pan‐acetyl‐lysine antibody as well as an anti‐HA antibody. β‐actin was used as a loading control. IP, immunoprecipitation. (I) ChIP‐qPCR analysis was used to determine the binding affinity of H3K27ac to the IRF7‐binding region in the promoter of ISGs including *IFIT1*, *OASL*, *ISG15*, and *IFITM3*. ChIP‐qPCR with IgG was performed as the control. Data shown in (B–D and I) are mean ± SD, ns *p* > .05, *p < .05, **p < .01, ****p < .0001 and statistical significance was assessed by a two‐tailed unpaired Student's *t* test.

As the transcription factor, IRF7 activation promotes transactivation of type I interferon and other ISGs.^8^ To study the role of ubiquitination on lysine 375 of IRF7 in host antiviral immune response, we transfected wild‐type or mutant IRF7‐K375R into HEK293T cells, which were subsequently infected with VSV‐GFP. As shown in Figure 6A,B, an increased percentage of GFP^+^ cells were detected in IRF7‐K375R‐expressing cells as compared with wild‐type IRF7‐expressing cells, indicating that the ubiquitination‐resistant mutation impaired the antiviral effects of IRF7. Accordingly, the transactivation activity of IRF7 was decreased when it bore the K375R mutation as compared with wild‐type IRF7 (Figure 6C,D). To investigate the mechanism by which ubiquitination‐resistant mutation restricted the transcriptional activity of IRF7, we first analyzed the subcellular localization of IRF7‐K375R with or without viral infection. Through nucleocytoplasmic separation and con‐focal assays, we found that this ubiquitination‐resistant mutation hardly affected the nuclear accumulation of IRF7 upon viral infection (Figure S5A,B). Considering the fact that K375R mutations are not located in the DNA‐binding domain of IRF7, we thus hypothesize that this ubiquitination‐resistant mutation may influence the co‐activator activity of IRF7. To this end, we transfected wild‐type IRF7 or IRF7‐K375R plasmid into HEK293T cells, which were subsequently infected with VSV for 8 h. Following the application of affinity purification with anti‐FLAG beads, the interactome of IRF7 or IRF7‐K375R was analyzed by mass spectrometry assay (Figure 6E). Among these IRF7 interactors, we noticed that IRF7‐K375R selectively interacted with HDAC1 that elicits epigenetic repressive effects, and plays an important role in transcriptional regulation (Figure 6F). Subsequent co‐immunoprecipitation assay further confirmed the results that IRF7^K375R^ strongly recruited HDAC1 (Figure 6G).

As a deacetylase, HDAC1 can affect the acetylation of both histone and nonhistone proteins. To determine whether HDAC1 promotes IRF7 deacetylation, we transfected the vectors encoding wild‐type or mutant IRF7‐K375R in the presence or absence of HDAC1 into HEK293T cells and analyzed the acetylation status of IRF7 by the anti‐acetylated lysine antibody. Following the immunoprecipitation by anti‐FLAG antibody, we found that the presence of HDAC1 hardly affected the acetylation of both wild‐type or mutant IRF7‐K375R (Figure 6H). We next used the anti‐H3K27ac antibody to assess the role of HDAC1 in the modulation of histone acetylation in cells transfected with vector encoding wild‐type or IRF7‐K375R. Through Chromatin immunoprecipitation‐qPCR (ChIP‐qPCR) assay, we found that overexpression of IRF7 increased the H3K27ac levels of the promoters of genes encoding IFIH1, IFITM3, ISG15, and OASL (Figure 6I), which is consistent with our data that the transcription of ISGs was increased in presence of IRF7 (Figure 6C,D). Conversely, overexpression of IRF7‐K375R inhibited the increase in H3K27ac levels to some extent (Figure 6I). In light of the role of NEURL3 in the modulation of IRF7 association with HDAC1, our data thus demonstrate that NEURL3 epigenetically modulated ISGs expression by augmenting the levels of H3K27ac on the IRF7‐binding regions.

### 3.7 Neurl3‐deficient mice exhibit increased susceptibility to viral infection

**FIGURE 7:**
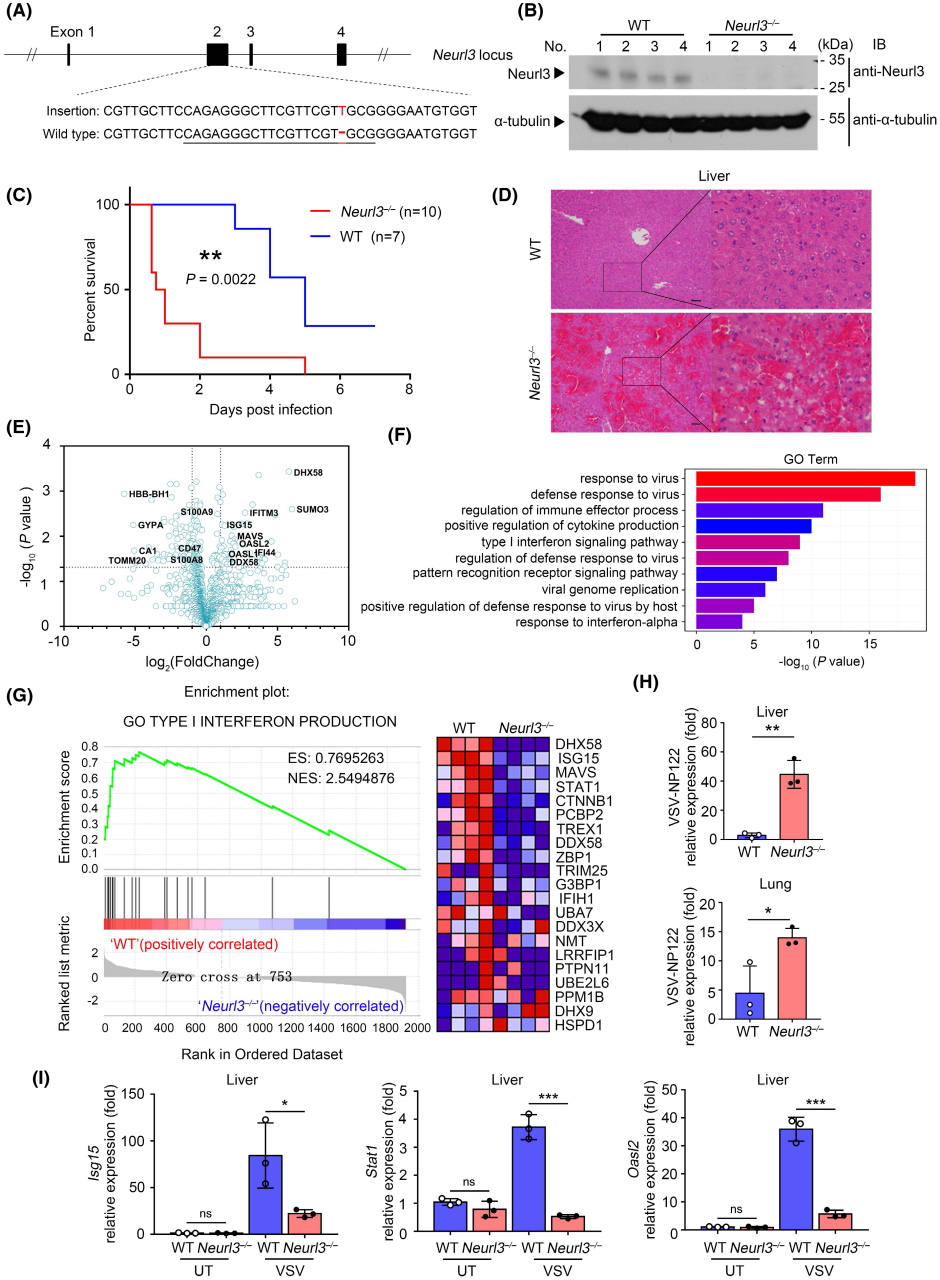
*Neurl3* ^*−/−*^ mice exhibit increased sensitivity to VSV‐induced inflammation. (A) Schematic representation of strategy for generation of *Neurl3* ^*−/−*^ mice by using CRISPR/Cas9 system with a T base insertion in exon2 of *Neurl3*. (B) Immunoblot analysis of NEURL3 protein in lungs from *Neurl3* ^*−/−*^ and WT mice (*n* = 4 mice per group). α‐tubulin was used as a loading control. (C) Survival analysis of *Neurl3* ^*−/−*^ mice (*n* = 10) and WT mice (*n* = 7) after intravenous injection of VSV (8 × 10^7^ PFU/mouse). (D) Hematoxylin–eosin staining of liver sections from WT and *Neurl3* ^*−/−*^ (*n* = 4 per group) mice infected with VSV (8 × 10^7^ PFU/mouse) for 24 h. Scale bars, 50 μm. (E) Volcano plots of differentially expressed genes in livers of WT and *Neurl3* ^*−/−*^ mice (*n* = 4 per group) infected with VSV (8 × 10^7^ PFU/mouse) for 24 h were subjected to MS analysis. (F) DAVID analysis by Gene Ontology (GO) terms of the proteins identified by MS that were significantly increased in the liver of WT and *Neurl3* ^*−/−*^ mice (*n* = 4 per group) infected with VSV (8 × 10^7^ PFU/mouse) for 24 h. (G) GSEA analysis of the differential proteins from livers of WT and *Neurl3* ^*−/−*^ mice (*n* = 4 per group) infected with VSV (8 × 10^7^ PFU/mouse) for 24 h and then measured by MS. ES, enrichment score. NES, normalized enrichment score. (H and I) The mRNA levels of VSV‐NP122 in livers and lungs as well as ISGs including *Isg15*, *Stat1*, and *Oasl2* in livers of WT and *Neurl3* ^*−/−*^ mice (*n* = 3 per group) infected with VSV (8 × 10^7^ PFU/mouse) or not for 24 h measured by qRT‐PCR. Data shown in (C) statistical significance was assessed by a Gehan–Breslow–Wilcoxon test. Data shown in (H and I) are mean ± SD, *p < .05, **p < .01, ***p < .001, ****p < .0001 and statistical significance was assessed by a two‐tailed unpaired Student's *t* test.

To further study the biological function of NEURL3 in vivo, we employed CRISPR‐Cas9 technology to generate *Neurl3* ^*−/−*^ mice with homo‐ or heterozygous frameshift mutation in exon2 of *Neurl3*, which introduced a pre‐stop codon into the open reading frame (ORF) of *Neurl3* (Figures 7A and S6A,B). These *Neurl3* ^*−/−*^ mice are fertile and develop normally. To confirm the fidelity and effectiveness of *Neurl3* ^*−/−*^ mice, we measured the endogenous NEURL3 expression in lungs from wild‐type (WT) and *Neurl3* ^*−/−*^ mice with anti‐NEURL3 antibody. As shown in Figure 7B, in contrast to the medium expression of NEURL3 in the lungs of WT mice, the expression of NEURL3 was undetectable in *Neurl3* ^*−/−*^ lung.

We next used VSV to infect WT and *Neurl3* ^*−/−*^ mice to evaluate the function of NEURL3 on the susceptibility of mice to viral infection. In accordance with the stimulatory effects of NEURL3 on host antiviral response, increased lethality was detected in *Neurl3* ^*−/−*^ mice as relative to their littermate controls post‐viral infection (Figure 7C). Gross tissue examination revealed that ecchymosis spots were detected in livers from *Neurl3* ^*−/−*^ mice (data not shown). We then used hematoxylin and eosin (H&E) staining and found that larger areas of necrosis were detected in livers from *Neurl3* ^*−/−*^ mice as compared with WT mice (Figure 7D). Moreover, we also employed the anti‐IRF7 antibody to pulldown the endogenous IRF7 and assessed the ubiquitin modification of IRF7 from livers in wild type or *Neurl3* ^*−/−*^ mice following viral infection. As shown in Figure S6C, the level of IRF7 ubiquitination was reduced in *Neurl3* ^*−/−*^ livers than that in wild type controls following viral infection, further confirming NEURL3 as the E3 ubiquitin ligase of IRF7.

To assess the antiviral immune response of *Neurl3* ^*−/−*^ mice, we performed a quantitative proteomic analysis between WT and *Neurl3* ^*−/−*^ livers. Physiologically, genes related to the lipid metabolic process and protein transportation were differentially expressed between WT and *Neurl3* ^*−/−*^ livers (Figure S6D). However, during viral infection, the expression of ISGs including DHX58, IFITM3, ISG15, and OASL was upregulated in the livers of WT mice, while *Neurl3* ^*−/−*^ livers expressed a higher level of proteins related to inflammatory response such as S100A9 and CD47 (Figure 7E). Using GO analysis, pathways including defense responses to virus and type I interferon signaling were enriched in livers of WT mice as compared with *Neurl3* ^*−/−*^ mice following viral infection (Figure 7F). Similar results were detected by GSEA analysis (Figures 7G and S6E). Consistent with the quantitative proteomic results, qRT‐PCR results showed that along with a lower level of viral titration, higher levels of ISGs such as *Isg15*, *Oasl2*, and *Stat1* were detected in livers of WT mice than those from *Neurl3* ^*−/−*^ mice post‐viral infection (Figure 7H,I). In addition to the liver, similar results were also detected in lungs from WT or *Neurl3* ^*−/−*^ mice post‐viral infection (Figures 7H and S6F). Collectively, our data demonstrate that the NEURL3‐IRF7 axis is an important positive signaling for the host innate immune response.

### 3.8 Activation of NF‐κB signaling is substantial for NEURL3 induction

**FIGURE 8:**
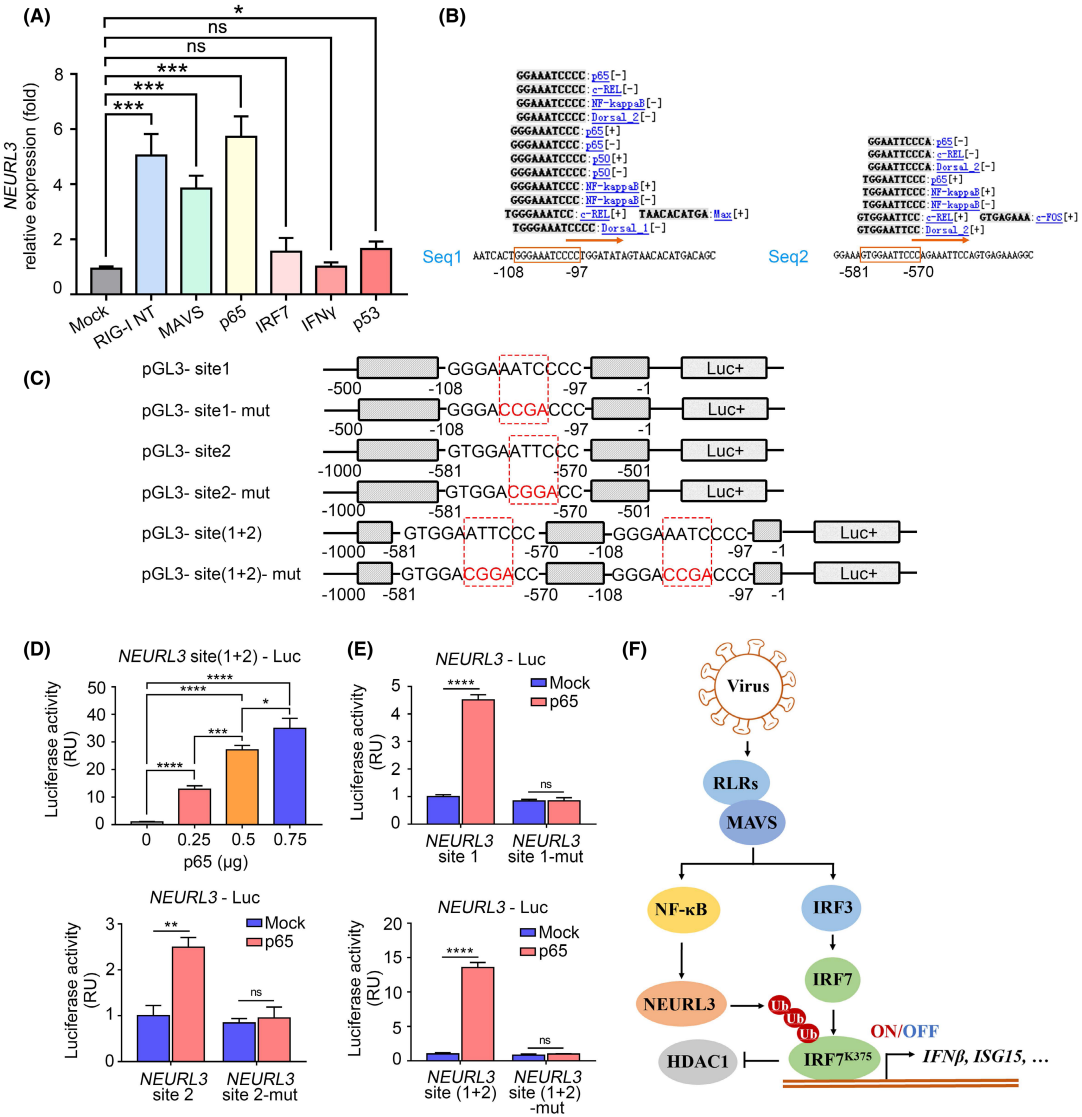
p65 is substantial for NEURL3 upregulation. (A) The mRNA level of *NEURL3* in HEK293T transfected with empty vector (Mock) or vector encoding RIG‐I NT, MAVS, p65, IRF7, IFN‐γ, or p53 for 24 h by qRT‐PCR analysis. (B) The binding sites prediction of *NEUEL3* promoter from Consite (http://consite.genereg.net/). (C) Schematic representation of *NEURL3* promoter containing site1 (−mut), site2 (−mut), and site (1 + 2) (−mut) constructed to pLG3 plasmid. p65 binding sites are circled by the red dotted line. (D) Luciferase (Luc) activity in HEK293T cells transfected with *NEURL3* containing site 1 and site 2 luciferase reporter together with plasmid encoding p65 in different doses (0, 0.25, 0.5, 0.75 μg) for 24 h. (E) Luciferase (Luc) activity in HEK293T cells transfected with different *NEURL3* luciferase reporter (The upper right, *NEURL3* site 1 and site 1 mut; the lower left, *NEURL3* site 2 and site 2 mut; the lower right, *NEURL3* site (1 + 2) and site (1 + 2) mut) together with plasmid encoding p65 or empty vector (Mock) for 24 h. (F) Model for the role of NEURL3 in host antiviral immune response. Data shown in (A, D, E) are mean ± SD, ns *p* > .05, *p < .05, **p < .01, ***p < .001, ****p < .0001 and statistical significance was assessed by a two‐tailed unpaired Student's *t* test.

In addition to viral infection, we found that activation of RIG‐I‐like (RLR) signaling (RIG‐I and MAVS) rather than IFN signaling contributed to the transactivation of *NEURL3* (Figure 8A). Furthermore, we noticed that p65, the core component of NF‐κB, selectively increased the expression of *NEURL3* (Figure 8A). Utilizing the Consite tool, we predicted the presence of two NF‐κB‐binding sites with the consensus sequence of (−108)GGGAAATCCCC(−97) and (−581)GTGGAATTCCC(−570) on the *NEURL3* promoter region (Figure 8B). We next cloned a series of wild‐type and mutant *NEURL3* promoter region sequence into the luciferase reporter vector (Figure 8C). As shown in Figure 8D, p65 increased the transcription of NEURL3 in a dose‐dependent manner. Conversely, mutation of NF‐κB‐binding sites dampened stimulatory effects of p65 on NEURL3 expression (Figure 8E). Our data thus demonstrate that p65 plays an important role in NEURL3 upregulation during viral infection.

Taken together, our data identify NEURL3 as an E3 ubiquitin ligase of IRF7. Through triggering K63‐linked poly‐ubiquitination of K375 on IRF7, NEURL3 impairs the association of IRF7 with HDAC1, consequently enhancing IRF7‐mediated transcription of type‐I interferon and other ISGs. Our study thus demonstrates that NEURL3 acts as an important positive regulator in the modulation of host antiviral immune response (Figure 8F).

## 4 DISCUSSION

IRF family members are critical transcriptional regulators in innate immune signaling and type I interferon production.^20^ Post‐translational regulations of IRF3 have profound impacts on host antiviral immunity,^21^ however, relatively little is known about the mechanism by which the biological function of IRF7 is modulated during viral infection. In the current study, we identified the E3 ubiquitin ligase NEURL3 as the positive regulator of IRF7 in host antiviral innate immune signaling. Although NEURL3 also interacted with IRF3 due to the high structural homology between IRF3 and IRF7, our study showed that NEURL3 is selectively upregulated in the late phase of host immune response, which is concomitant with IRF7 induction. It is conceivable that NEURL3 exerts preferential effects on IRF7 rather than IRF3 upon exposure to the virus. In addition to viral infection, NEURL3 can be induced in the alveolar epithelial cells upon exposure to LPS.^22^ In light of the interaction between NEURL3 and IRF3 or IRF7, we thus hypothesize that NEURL3 can also affect inflammation through the regulation of interferon signaling during bacterial infection.

Consistent with our study, another group has found that loss of NEURL3 elicits stimulatory effects on HCV infection through targeting HCV glycoprotein E1.^16^ However, the biological function of NEURL3 in vivo remains elusive due to the lack of *Neurl3* knockout mouse model. In our present study, we employed CRISPR‐Cas9 technology to generate *Neurl3* ^*−/−*^ mice with homo‐ or heterozygous frameshift mutation in exon2 of *Neurl3*, which introduced a pre‐stop codon into the open reading frame (ORF) of *Neurl3*. These mice are fertile and develop normally. More importantly, the *Neurl3* ^*−/−*^ mice exhibited increased susceptibility to viral infection, which was assessed by survival curve, histological analysis, and proteomic study. Our data thus uncover the stimulatory role of NEURL3 in host innate immune response and provide a new potential target for antiviral treatment.

As one of the most abundant post‐translational modifications in mammalian cells, ubiquitination can trigger targeted protein degradation (mainly K48‐linked ubiquitination) or activate the signal transduction (mainly K63‐linked ubiquitination).^23^ In this study, we found that NEURL3 triggered K63‐linked poly‐ubiquitination on IRF7. Moreover, NEURL3‐mediated ubiquitin modification was specifically on the lysine 375 of IRF7, which is highly conserved in multiple species. In accordance with our data, the ubiquitination of IRF7 lysine 375 was also recorded in the PhosphoSitePlus database (www.phosphosite.org/),^24^ while its function in IRF7‐mediated immune response was unknown. Our data are thus the first, to our knowledge, to identify the ubiquitination of IRF7 lysine 375 that promotes the transcriptional activity of IRF7 by disrupting the association of IRF7 with HDAC1, consequently augmenting the host antiviral immune response.

Although NEURL3 acts as a putative E3 ubiquitin ligase, its substrates are largely unknown. In this study, we identified that NEURL3 physically interacted with IRF7 and modulated IRF7 activity in an E3 ligase‐dependent manner. Interestingly, our data predicted that the C217Y mutation in the RING domain of NEURL3 would inactivate its ligase function. As expected, this mutant NEURL3^C217Y^ elicited little effects on IRF7‐mediated immune response, similarly to NEURL3 without the RING domain. Notably, C217Y mutation is detected in patients with endometrial cancer.^25^ In light of the essential role of C217 in NEURL3‐mediated innate immune response, it is conceivable that the C217Y mutation can impair host antitumor immune response and facilitate cancer development. New attempts should focus on the role of NEURL3 in the modulation of cancer immune escape.

In conclusion, our study identifies NEURL3, as an E3 ubiquitin ligase of IRF7 that specifically triggers K63‐linked poly‐ubiquitination on IRF7 lysine 375 and interrupts its association with HDAC1. The dissociation of IRF7 with HDAC1 then enhances the H3K27ac signals on the IRF7‐binding region, which enhances the transcription of ISGs, consequently augmenting the host antiviral immune response. Accordingly, *Neurl3* ^*−/−*^ mice produce less type I IFNs and exhibit increased susceptibility to viral infection. These findings, therefore, provide new insights into the role of the NEURL3 in the regulation of the host innate immune response against viral infection.

## AUTHOR CONTRIBUTIONS

Fang Qi and Dan Lu conceived and designed the experiments; Fang Qi performed most of the experiments and analyzed the data; Xin Zhang, Caixia Ren and Jianyuan Luo assisted in some experiments; Likun Wang and Xuyang Zhao performed mass spectrometry analysis; Dan Lu, Fang Qi and Jianyuan Luo wrote the paper.

## DISCLOSURES

The authors declare no competing financial interests in relation to the study described.

## Supporting information


Fig S1‐S6

Table S1‐S2

## Data Availability

The data that support the findings of this study are available in the methods and/or supplemental material of this article.
