# Supplementary material for: E3 ubiquitin ligase NEURL3 promotes innate antiviral response through catalyzing K63‐linked ubiquitination of IRF7
Source: FASEB J. 2022 Jul 6;36(8):e22409. doi: 10.1096/fj.202200316R (PMC12166272; doi:10.1096/fj.202200316R)
Supplement: Supplementary file 1 — Fig S1‐S6 [file FSB2-36-e22409-s001.docx]

**SUPPLEMENTAL FIGURES**

**
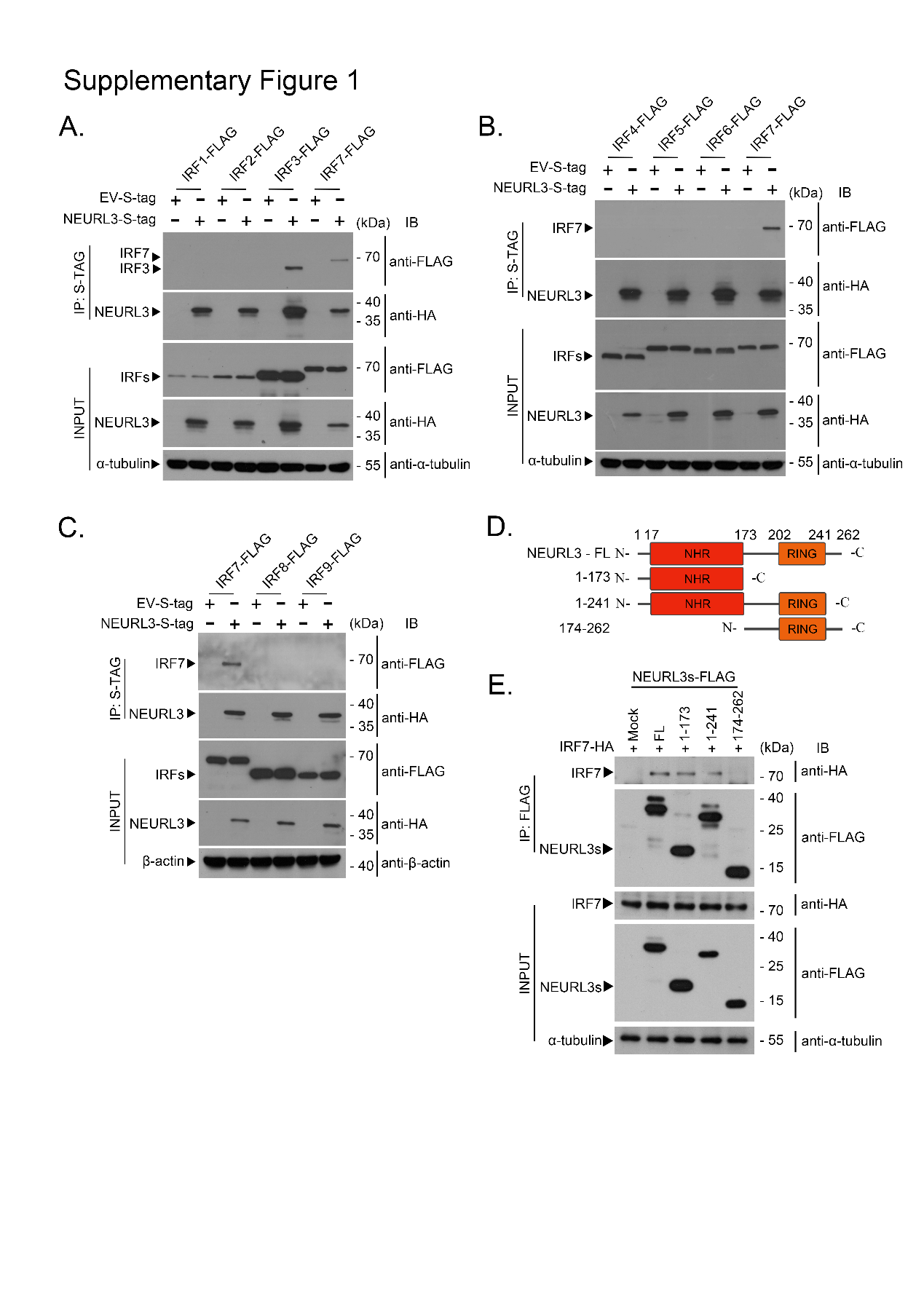
**

**Supplementary Figure 1, related to Figure 1. The relationship of NEURL3 with IRF family members.**

(A-C) Coimmunoprecipitation analysis of HEK293T cells co-transfected vectors encoding FLAG-IRFs (A, IRF1, IRF2, IRF3, IRF7; B, IRF4, IRF5, IRF6, IRF7; C, IRF7, IRF8, IRF9) in presence or absence of S-TAG-HA-NEURL3; lysates immunoprecipitated with S-TAG beads were analyzed by immunoblot with anti-FLAG and anti-HA antibodies, respectively. α-tubulin was used as loading control. IP, immunoprecipitation.

(D) Schematic representation of full length NEURL3 and NEURL3 truncation mutants. NEURL3-1-173 (from N terminal to NHR domain), NEURL3-1-241 (C terminal linker deleted), NEURL3-174-262 (N terminal linker and NHR domain deleted).

(E) Immunoassay of HEK293T cells transfected with FLAG-NEURL3 truncation mutants or empty vector and HA-IRF7; lysates immunoprecipitated with anti-FLAG antibody were analyzed by immunoblot with anti-HA antibody. α-tubulin was used as loading control. Mock, empty vector; FL, full length NEURL3; 1-173, NEURL3 of 1-173 amino acid; 1-241, NEURL3 of 1-241 amino acid; 174-262, NEURL3 of 174-262 amino acid.


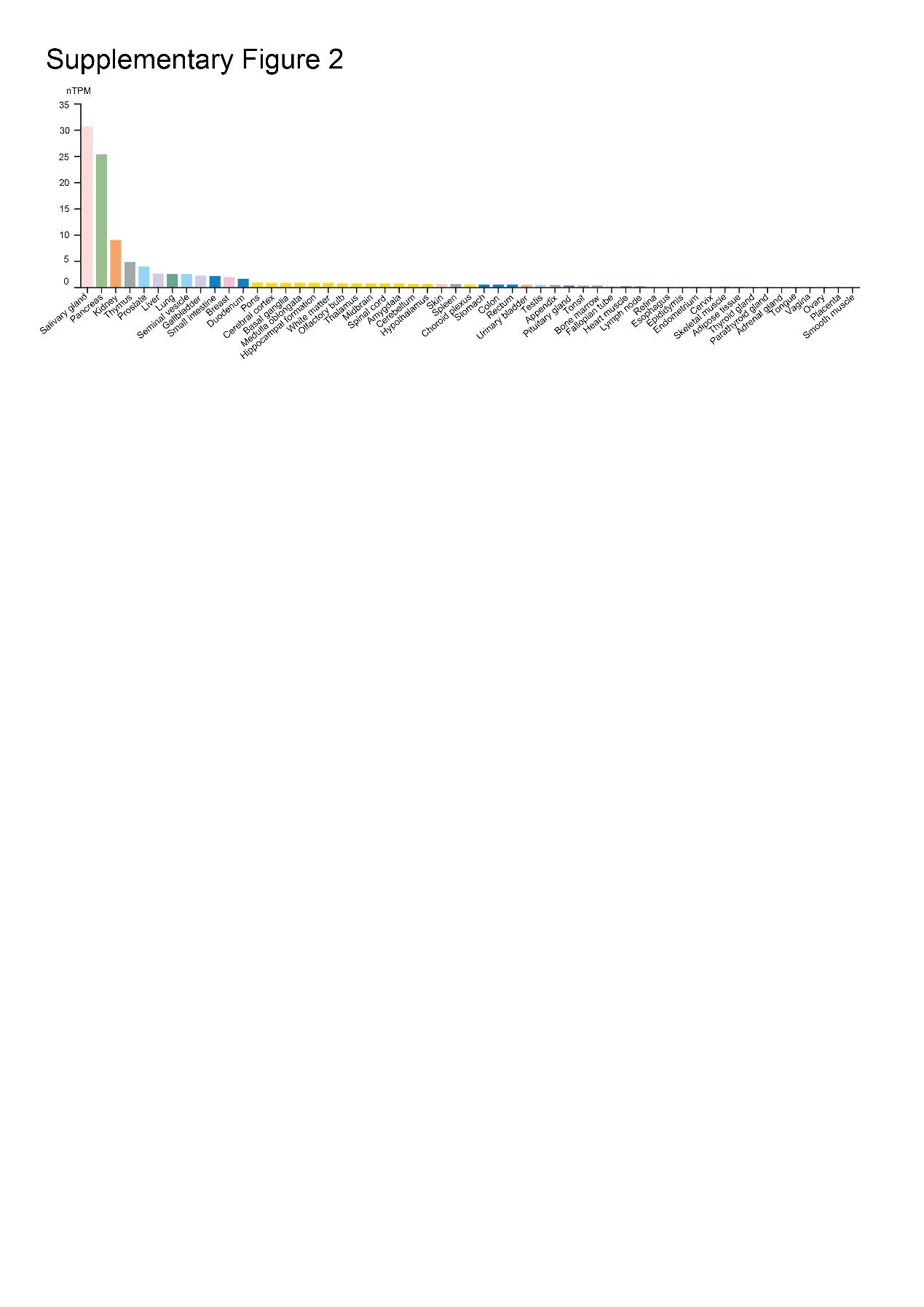


**Supplementary Figure 2, related to Figure 2. NEURL3 expression profiling.**

*NEURL3* expression pattern in Consensus dataset from THE HUMAN PROTEIN ATLAS.


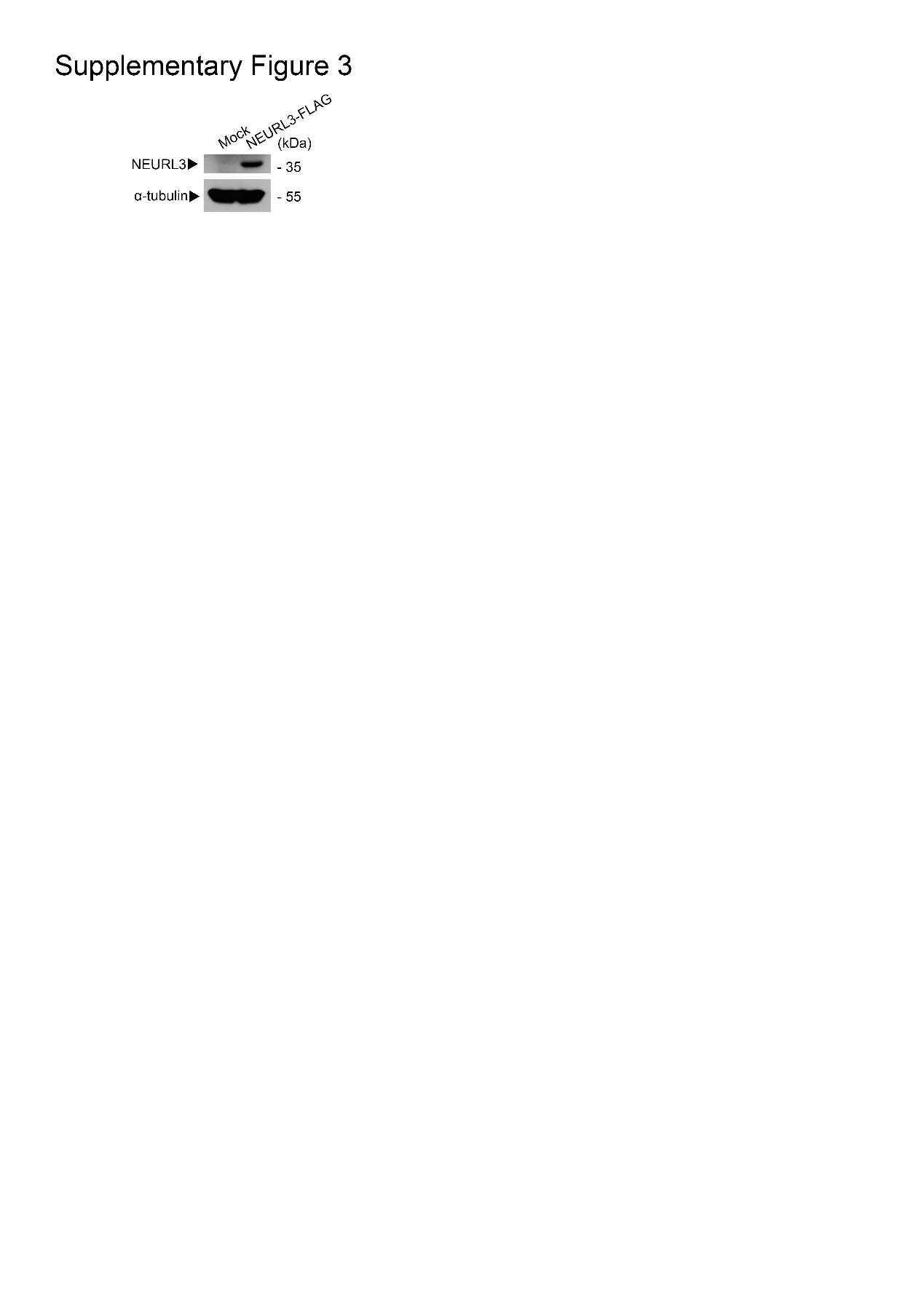


**Supplementary Figure 3, related to Figure 3. Immunoblot analysis of H1299 cell lines stably expressing NEURL3 or empty vector.** α-tubulin was used as loading control.


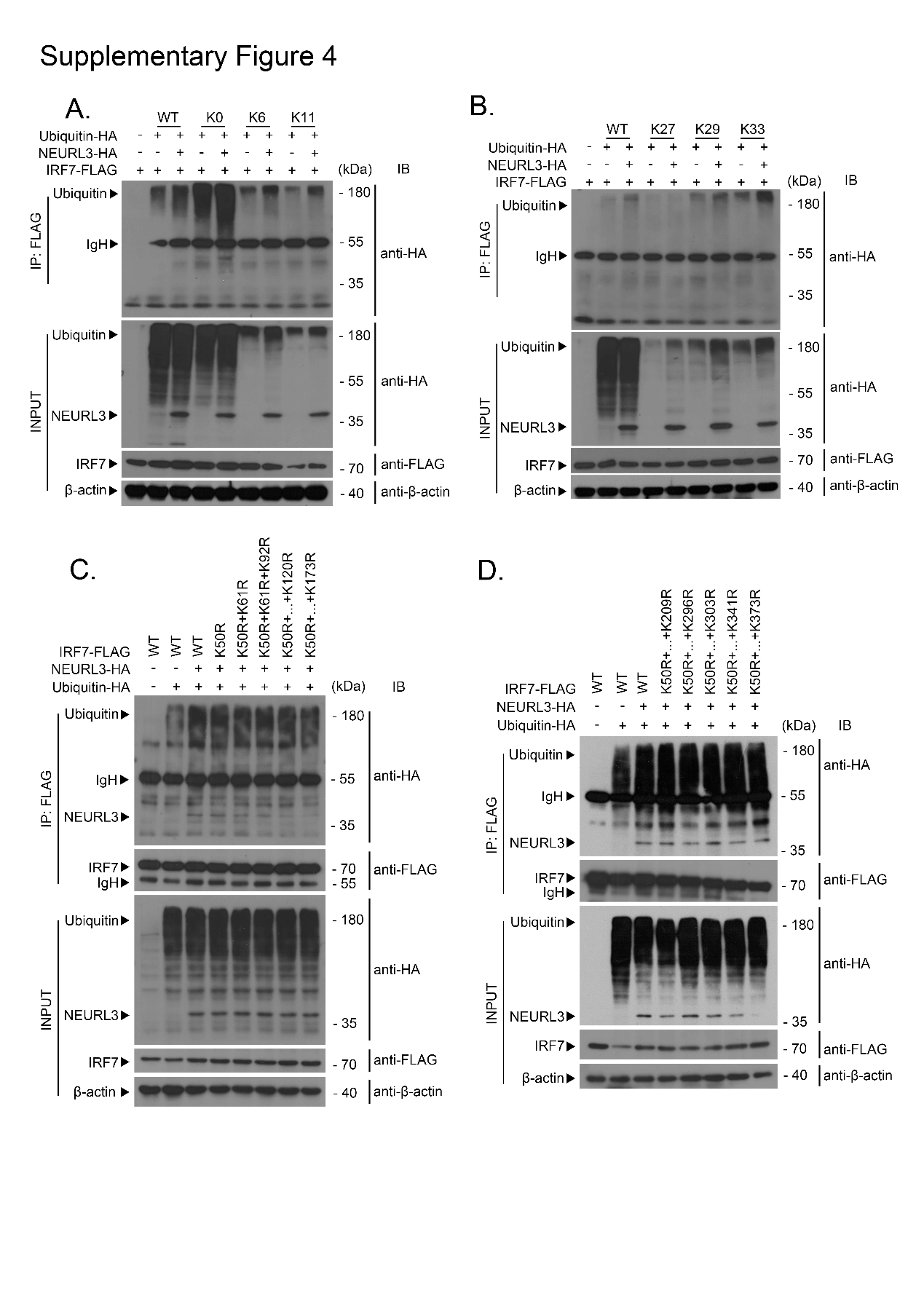


**Supplementary Figure 4, related to Figure 5. NEURL3 promotes K63-linked ubiquitination of IRF7 at K375.**

(A) Coimmunoprecipitation analysis of IRF7 ubiquitination in HEK293T transfected with FLAG-IRF7, HA-NEURL3 or empty vector and HA-ubiquitin-WT, HA-ubiquitin-K0, HA-ubiquitin-K6 or HA-ubiqutin-K11. Lysates immunoprecipitated with anti-FLAG antibody were analyzed by immunoblot with anti-HA antibody. β-actin was used as loading control. IP, immunoprecipitation.

(B) Coimmunoprecipitation analysis of IRF7 ubiquitination in HEK293T transfected with FLAG-IRF7, HA-NEURL3 or empty vector and HA-ubiquitin-WT, HA-ubiquitin-K27, HA-ubiquitin-K29 or HA-ubiqutin-K33. Lysates immunoprecipitated with anti-FLAG antibody were analyzed by immunoblot with anti-HA antibody. β-actin was used as loading control. IP, immunoprecipitation.

(C) Coimmunoprecipitation analysis of IRF7 ubiquitination in HEK293T transfected with HA-NEURL3 or empty vector, HA-ubiquitin and FLAG-IRF7-WT, FLAG-IRF7-K50R, FLAG-IRF7-K50R+K61R, FLAG-IRF7-K50R+K61R+K92R, FLAG-IRF7-K50R+…+K120R, FLAG-IRF7-K50R+…+K173R. Lysates immunoprecipitated with anti-FLAG antibody were analyzed by immunoblot with anti-HA antibody. β-actin was used as loading control. IP, immunoprecipitation.

(D) Coimmunoprecipitation analysis of IRF7 ubiquitination in HEK293T transfected with HA-NEURL3 or empty vector, HA-ubiquitin and FLAG-IRF7-WT, FLAG-IRF7-K50R+…+K209R, FLAG-IRF7-K50R+…+K296R, FLAG-IRF7-K50R+…+K303R, FLAG-IRF7-K50R+…+K341R, FLAG-IRF7-K50R+…+K373R. Lysates immunoprecipitated with anti-FLAG antibody were analyzed by immunoblot with anti-HA antibody. β-actin was used as loading control. IP, immunoprecipitation.


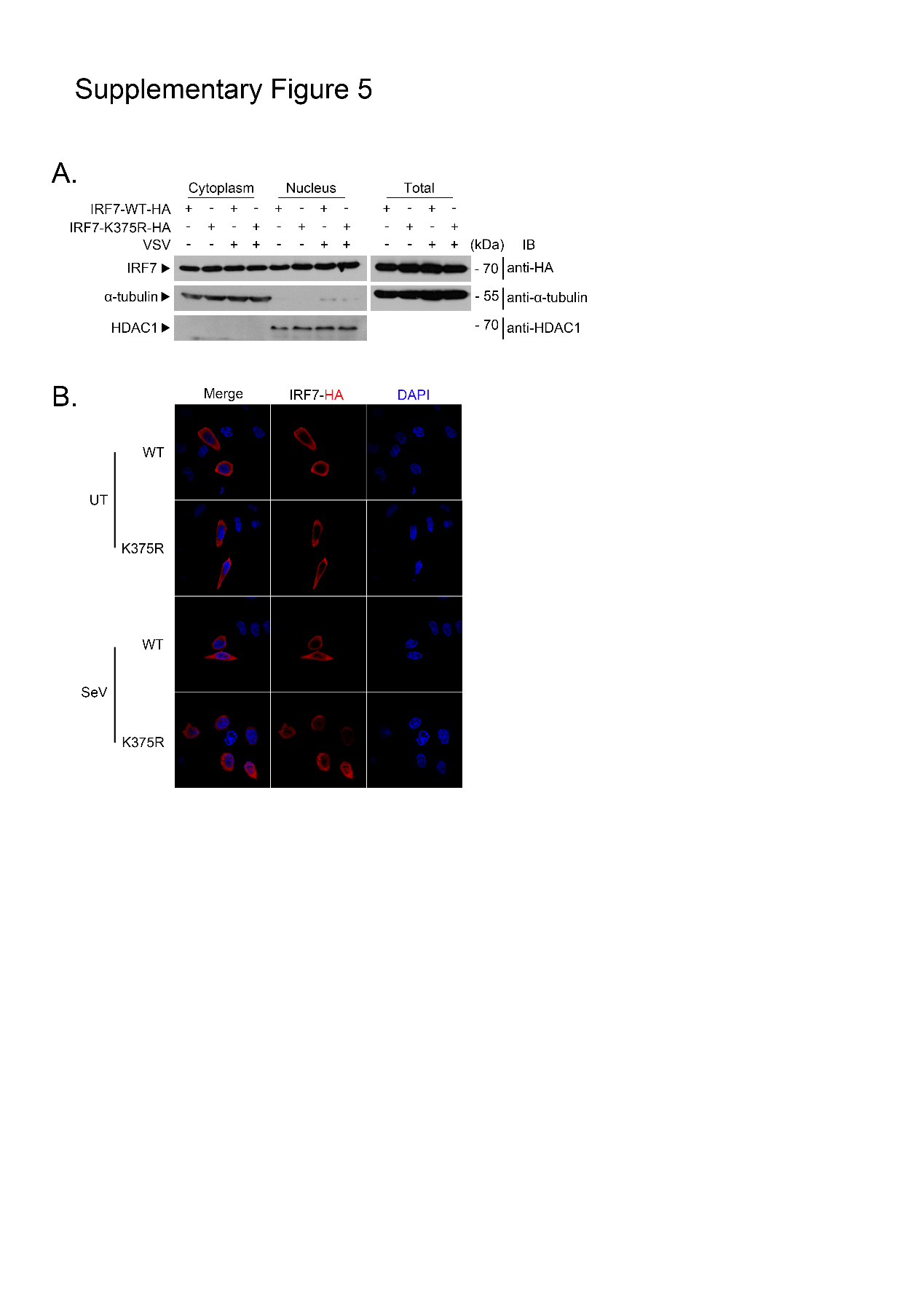


**Supplementary Figure 5, related to Figure 6. NEURL3 exerts little effects on the subcellular localization of IRF7.**

(A) Immunoblot analysis of HA-IRF7 in total, nuclear and cytoplasmic fractions in HEK293T cells transfected with HA-IRF7-WT/K375R, followed by VSV infection for 24 hrs. α-tubulin served as cytoplasmic control. HDAC1 served as nuclear protein control.

(B) Anti-HA specific immunostaining of Hela cells were transfected with HA-IRF7-WT or HA-IRF7-K375R after SeV infection for 24 hrs. HA-IRF7 was shown by confocal fluorescence microscopy. DAPI was used to indicate the nucleus. UT, untreatment. Scale bars, 10 μm.


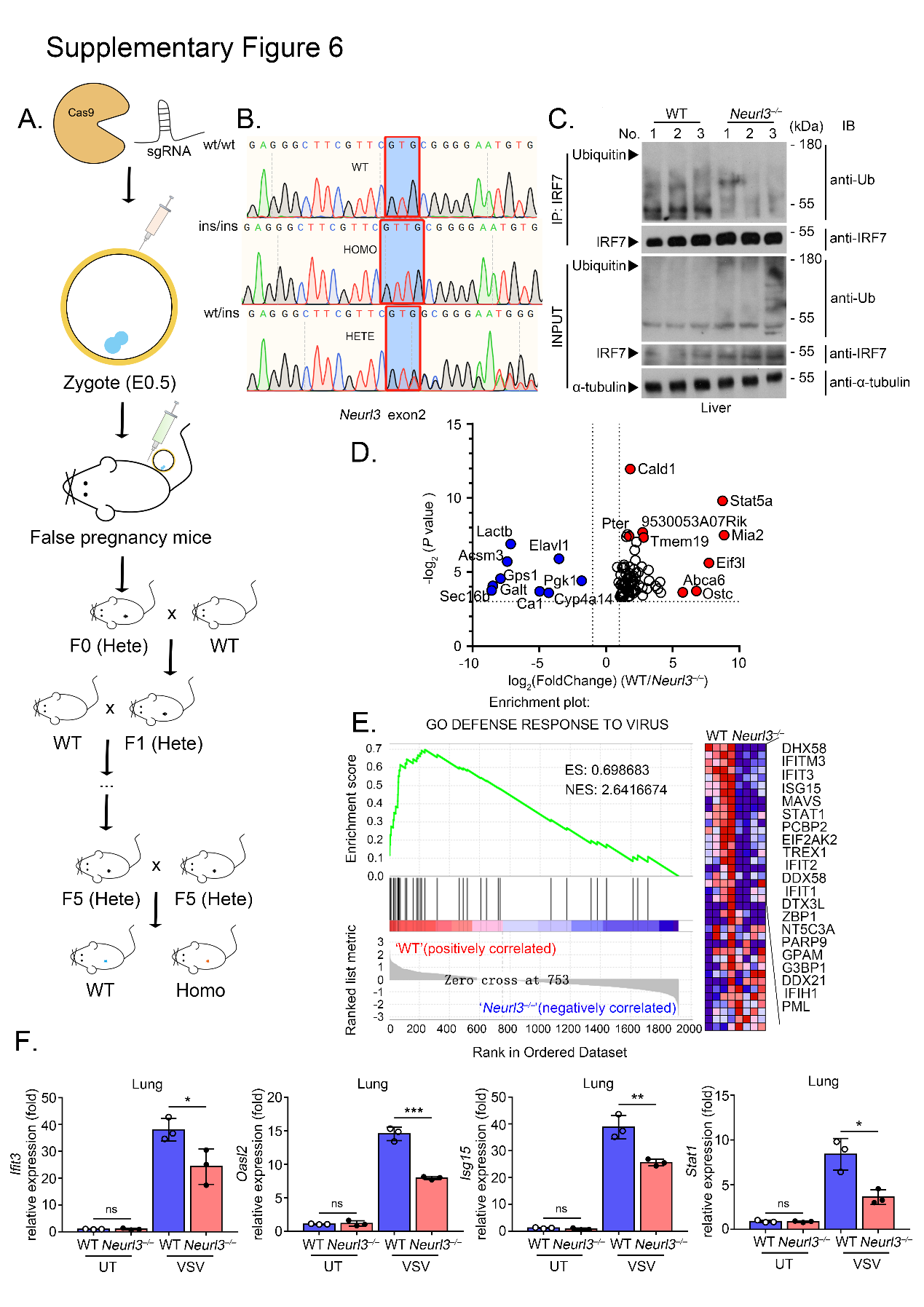


**Supplementary Figure 6, related to Figure 7. *Neurl3^–/–^* mice exhibit impaired antiviral immune response.**

1. Generation of *Neurl3^–/–^* mice.
2. The sequencing diagram of exon2 in WT, *Neurl3^+/–^*, *Neurl3^–/–^* mice.

(C) Coimmunoprecipitation analysis of endogenous IRF7 ubiquitination in virus-infected WT and *Neurl3^–/–^* livers. Lysates immunoprecipitated with anti-IRF7 antibody were analyzed by immunoblot with anti-ubiquitin antibody. α-tubulin was used as loading control. IP, immunoprecipitation.

(D) Volcano plots of differentially expressed genes in livers of WT and *Neurl3^–/–^* mice without viral infection (n = 3 per group) were subjected to MS analysis.

(E) Livers of WT and *Neurl3^–/–^* mice (n = 4 per group) infected with VSV (8 × 10^7^ PFU/mouse) for 24 hrs were subjected to MS analysis. The differential proteins were analyzed using GSEA with GO gene sets. ES, enrichment score. NES, normalized enrichment score.

(F) The mRNA levels of ISGs including *Ifit3,* *Oasl2, Isg15 and Stat1* in lungs of WT and *Neurl3^–/–^* mice (n = 3 per group) infected with VSV (8 × 10^7^ PFU/mouse) or not for 24 hrs measured by qRT-PCR.

Data shown in (F) are mean ± SD, **P* < 0.05, ***P* < 0.01, ****P* < 0.001, *****P* < 0.0001 and statistical significance was assessed by a two-tailed unpaired Student’s t test.
