# Supplementary material for: E3 ubiquitin ligase NEURL3 promotes innate antiviral response through catalyzing K63‐linked ubiquitination of IRF7
Source: FASEB J. 2022 Jul 6;36(8):e22409. doi: 10.1096/fj.202200316R (PMC12166272; doi:10.1096/fj.202200316R)
Supplement: Supplementary file 2 — Table S1‐S2 [file FSB2-36-e22409-s002.doc]

**SUPPLEMENTAL TABLE**

**Table S1:** List of primers for qRT-PCR analysis in this study.

| Primer name | Sequence (5'->3') |
| --- | --- |
| *GAPDH* | ACCCACTCCTCCACCTTTGA |
|  | CTGTTGCTGTAGCCAAATTCGT |
| *NEURL3* | GACGTGTATGGGACCACTAAG |
|  | TAGAAGCAGATGGCACACTC |
| *CXCL10* | TGCCATTCTGATTTGCTGCC |
|  | TGATGGCCTTCGATTCTGGA |
| *ISG20* | CCTACACAAGAGCATCCAGAAC |
|  | CTCGGATTCTCTGGGAGATTTG |
| *ISG56* | CCACAAGACAGAATAGCCAGAT |
|  | GTACTCATGGTTGCTGTAAATTAGG |
| *Actb* | GAGACCTTCAACACCCCAGC |
|  | ATGTCACGCACGATTTCCC |
| *Neurl3* | CCTTCACAGACATCTTCAGACC |
|  | GCGTTCCCGTGGAAACTAA |
| *Isg15* | GCCAGAAGCAGACTCCTTAAT |
|  | ACACCAGGAAATCGTTACCC |
| *Oasl2* | GATGGATATCCTCCCAGCTTAC |
|  | CAGGGTAGCCCTTACTTCTTATC |
| *Stat1* | CTGGCCCTGATGGTCTTATTC |
|  | GAGCTCTAGGATGGTGTCAATC |
| *Ifit3* | CCTCAGAACCAGTACCTGAAAG |
|  | TGGAGGACATCCGTTTGATTAG |
| VSV-NP122 | TGTATCCTTGAAAGCTCTGGAC |
|  | GTGTTCTGCCCACTCTGTATAA |

**Table S2:** List for primers for ChIP-qPCR analysis in this study.

| Primer name | Sequence (5'->3') |
| --- | --- |
| *OASL* | GTGAGGGTCAAAGAGGTTAGAG |
|  | GAAGGGACTCACTAGTGTTCAG |
| *ISG15* | TTCCCTGTCTTTCGGTCATTC |
|  | ATTTGGCTTCAGTTTCGGTTTC |
| *IFITM3* | CCCACACCACTAACAAGATGAG |
|  | ACTTTAGGAGAGGGAGGAAAGA |
| *IFIT1* | CACCATTGGCTGCTGTTTAG |
|  | CTCCTCTGAGATCTGGCTATTC |
